# Pathologically responsive ZnSrMo-LDH/Cu nanozymes with cascade antioxidant and angiogenic functions for myocardial ischemia-reperfusion treatment

**DOI:** 10.7150/thno.118420

**Published:** 2026-01-01

**Authors:** Jian Xu, Susu Zhang, Yu Yang, Xingwei Wei, Yunteng Fang, Zhilin Wang, Linwen Lan, Jiayi Shen, Enqian Liu, Wuming Hu, Tingting Hu, Chaojie Yu, Ruizheng Liang, Lingchun Lyu

**Affiliations:** 1 Department of Cardiology, The Fifth Affiliated Hospital of Wenzhou Medical University, Lishui Central Hospital, Lishui, 323000, P. R. China.; 2 State Key Laboratory of Chemical Resource Engineering, Beijing Advanced Innovation Center for Soft Matter Science and Engineering, Beijing University of Chemical Technology, Beijing 100029, P. R. China.; 3 Department of Electrical Engineering, City University of Hong Kong, 83 Tat Chee Ave, Kowloon Tong, Hong Kong SAR 999077, P. R. China.; 4 Henan Provincial People's Hospital, People's Hospital of Zhengzhou University, Zhengzhou 450003, P. R. China.; 5 Quzhou Institute for Innovation in Resource Chemical Engineering, Quzhou 324000, P. R. China.

**Keywords:** myocardial ischemia/reperfusion, antioxidation, nanozymes, angiogenesis, layered double hydroxides

## Abstract

**Rationale:** Myocardial ischemia-reperfusion (MI/R) injury induces apoptosis, metabolic dysregulation, and ventricular remodeling through complex pathological mechanisms. Although nanozyme engineering has the potential for antioxidation, reoxygenation, and pro-vascularization, achieving responsive modulation of the pathological microenvironment remains significantly challenging.

**Methods:** A layered double hydroxide (LDH)-based nanozyme (ZnSrMo-LDH/Cu) was synthesized via a low-temperature hydrothermal/isomorphic substitution method for MI/R treatment. The reactive oxygen species (ROS) scavenging ability and responsive ion release performance of ZnSrMo-LDH/Cu were evaluated through various spectroscopic characterization methods. The biosafety and therapeutic efficiency of ZnSrMo-LDH/Cu-BSA nanozymes were assessed by *in vitro* and *in vivo* experiments.

**Results:** ZnSrMo-LDH/Cu demonstrated cascade superoxide dismutase (SOD) and catalase (CAT) activities, effectively overcoming acidic microenvironment limitations to maintain CAT activity rather than peroxidase (POD) activity while scavenging ROS to generate oxygen, with a ROS scavenging capacity 2.97 times that of Fe_3_O_4_. Moreover, the acid-triggered Sr^2+^ release promoted vascular regeneration and synergistically improved the ischemic-hypoxic microenvironment. Consequently, after bovine serum albumin (BSA) modification, ZnSrMo-LDH/Cu-BSA demonstrated excellent cytoprotective effects, reducing the cardiomyocyte apoptosis rates to 9.4% (*in vitro*) and 20.7% (*in vivo*) of the levels in the MI/R group. *In vivo* studies further validated that ZnSrMo-LDH/Cu-BSA enhanced cardiac function and attenuated ventricular remodeling by inhibiting oxidative stress and promoting angiogenesis. Mechanistically, ZnSrMo-LDH/Cu-BSA provided a cardioprotective effect by inhibiting the TGF-β signaling pathway, thereby alleviating cell damage caused by MI/R.

**Conclusions:** The pathologically responsive LDH-based nanozyme represents a promising avenue for MI/R treatment.

## Introduction

Myocardial infarction (MI), caused by ischemic arterial occlusion, represents a leading global cause of mortality due to its acute onset, severe complications, and high lethality 1. Statistically, cardiovascular diseases collectively account for approximately one-third of deaths worldwide, with an estimated 50 million individuals affected by MI annually 2. Currently, the cornerstone therapeutic intervention for MI in clinical management remains prompt reperfusion. However, while reperfusion therapies can mitigate acute cardiovascular complications by timely restoring blood flow, the subsequent reoxygenation process generates excessive reactive oxygen species (ROS) in the ischemic myocardium, inadvertently aggravating tissue injury. This myocardial ischemia/reperfusion (MI/R) injury triggers irreversible cardiomyocyte apoptosis, fibrotic remodeling, and impaired cardiac function, ultimately increasing the risk of life-threatening sequelae such as heart failure 3. The MI/R microenvironment is characterized by three interdependent pathological features, including cellular hypoxic-ischemic injury, ROS-mediated oxidative stress, and tissue acidification due to lactate accumulation 4,5. These factors disrupt redox homeostasis, activate apoptotic cascades, and exacerbate cardiac dysfunction, rendering MI/R treatment exceptionally challenging 6. Although conventional antioxidant pharmacotherapy has demonstrated partial efficacy in managing MI/R injury, there are still therapeutic limitations that fail to dynamically modulate the pathological microenvironment 7,8. Therefore, achieving synchronized restoration of ROS levels, pH balance, oxygen tension, and vascularization to physiological homeostasis remains a formidable challenge in current therapeutic strategies.

The ROS burst during MI/R represents a primary pathogenic factor and originates from mitochondrial dysfunction, characterized by electron transport chain impairment and diminished oxidative phosphorylation activity 9,10. Consequently, ROS scavenging has become a key therapeutic strategy for MI/R injury 11,12. Although several natural antioxidants have been used for the treatment of MI/R injury, such as salvianolic acid B, curcumin, coenzyme Q, and resveratrol, these small molecule drugs generally have drawbacks, including prolonged onset of action, short circulation time, and limited efficacy 13,14. These limitations have driven significant research efforts toward developing artificial antioxidant enzyme mimics 15-17. Among them, copper-based nanozymes have shown particular prospects as superoxide dismutase (SOD)/catalase (CAT) mimics, demonstrating superior electron transfer efficiency and ROS scavenging capacity 18. However, the currently reported nanozymes convert from CAT activity to deleterious peroxidase (POD) activity in acidic microenvironments due to lactate accumulation, resulting in decreased or failed ROS scavenging ability 19,20. Moreover, the enhanced POD activity leads to the further conversion of SOD-catalyzed H_2_O_2_ production into highly toxic hydroxyl radicals (·OH), posing a potential risk to the therapeutic process 21,22. Therefore, it remains a formidable challenge to develop biomimetic nanozymes that can simultaneously amplify ROS scavenging activity, alleviate tissue hypoxia and reverse the pH imbalance in the microenvironment.

Pro-angiogenic regeneration represents another pivotal strategy in MI/R treatment by rapidly restoring myocardial perfusion and reestablishing collateral circulation. Revascularization of the infarcted myocardium is essential for delivering oxygen and nutrients to compromised cardiomyocytes, thereby preventing cellular necrosis and facilitating functional recovery 23,24. Recent studies have shown that bioactive metal ions such as Sr^2+^, Si^4+^, and Eu^3+^ can regulate vascular regeneration 25-29. Particularly noteworthy is the demonstrated angiogenic potential of Sr^2+^, which can enhance neovascularization in the ischemic myocardium while mitigating functional impairment and fibrotic scarring 30. However, the currently available pro-angiogenic biomaterials exhibit limited responsiveness in releasing bioactive ions under acidic microenvironment and lack the capacity to neutralize ischemic acidic microenvironment, thereby constraining their therapeutic efficacy in myocardial injury repair. This highlights an urgent demand for developing intelligent nanozymes with integrated pH-responsive properties and acid-neutralizing functionality. Such innovative systems will enable spatiotemporally controlled ion delivery in response to the acidic microenvironment to accelerate angiogenesis, while enhancing ROS scavenging capacity by strategic reversal of lactate accumulation and subsequent limitation of POD activity. Therefore, the development of intelligent nanozymes with combined pH-responsive behavior, tissue acidification reversion, efficient ROS elimination, and pro-angiogenic activity will be a promising strategy for MI/R injury.

**Figure 1:**
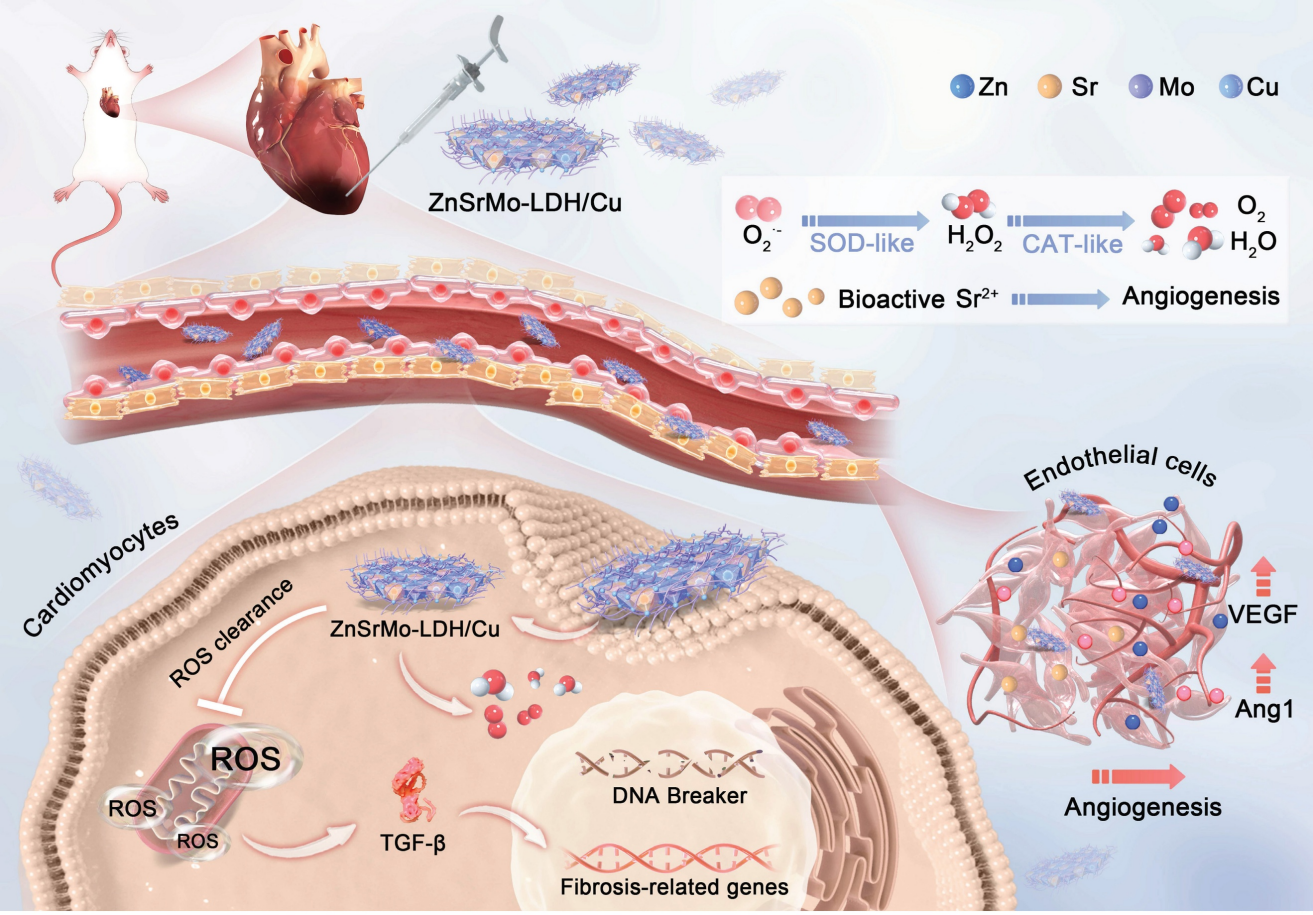
Schematic illustration of the preparation of ZnSrMo-LDH/Cu-BSA nanosheets and their application for MI/R injury treatment. ZnSrMo-LDH/Cu-BSA nanosheets not only demonstrate superior efficacy in scavenging ROS but also exhibit acid-responsive release of Sr^2+^ for revascularization. As a result, ZnSrMo-LDH/Cu-BSA nanosheets provide excellent cardioprotective effects by modulating the TGF-β signaling pathway, thereby alleviating myocardial injury caused by MI/R stimulation.

Layered double hydroxides (LDHs) are uniquely suited for adaptive modulation of MI/R microenvironments due to their biocompatibility, biodegradability, pH responsiveness, and highly tunable ionic composition 31-34. Their large specific surface area and abundant active sites facilitate efficient loading of antioxidant agents, as exemplified by astaxanthin-loaded MgAl-LDH, which enables microenvironment-responsive antioxidant release 35. Additionally, enzyme-like catalytic activities can be engineered through rational selection of lamellar metal ions. For example, LDHs containing redox-active metal ions such as (Co^2+^/Co^3+^, Mo^5+^/Mo^6+^, Cu^+^/Cu^2+^) exhibit SOD-like activity 36. The pathology-responsive LDH nanozymes enable a myocardial repair strategy that combines acidic microenvironment modulation with ROS scavenging. Herein, we develop ZnSrMo-LDH/Cu nanozymes by integrating multiple bioactive ions through an isomorphic substitution strategy for MI/R treatment (Figure 1). Importantly, acid-neutralization and pH-responsive ion release make ZnSrMo-LDH/Cu an ideal candidate for developing precise ROS scavengers and responsive pro-angiogenesis therapies. Specifically, ZnSrMo-LDH/Cu can enhance SOD and CAT activities in acidic microenvironments, converting excess superoxide radicals (·O_2_^-^) into O_2_ and H_2_O through cascade reactions, with a ROS scavenging ability 2.97 times that of Fe_3_O_4_. Moreover, ZnSrMo-LDH/Cu can respond to acidic microenvironments to release bioactive metal ions and further reverse tissue acidification, promoting vascular regeneration while avoiding the risk of harmful POD activity. *In vitro* and *in vivo* experiments demonstrate that after bovine serum albumin (BSA) modification, ZnSrMo-LDH/Cu-BSA reduces cardiomyocyte apoptosis rates to 9.4% (*in vitro*) and 20.7% (*in vivo*) of the levels in the MI/R group by clearing ROS and mitigating oxidative damage from oxygen-glucose deprivation/reperfusion (OGD/R). More importantly, ZnSrMo-LDH/Cu-BSA significantly promotes neovascularization, reduces infarct size, and improves cardiac function. Mechanistically, transcriptomic and proteomic analyses indicate that ZnSrMo-LDH/Cu-BSA performs various biological functions, particularly inhibiting the TGF-β signaling pathway to provide cardioprotective effects. Overall, this study introduces a nanoreactor with cascading antioxidant enzyme-like properties and angiogenic characteristics to simultaneously overcome acidification, ROS, hypoxia, and ischemic microenvironments, offering a promising therapeutic strategy for MI/R injury.

## Materials and Methods

### Materials

Zinc nitrate hexahydrate (Zn(NO_3_)_2_·6H_2_O, >99.0%), strontium nitrate (Sr(NO_3_)_2_, >99.0%), ammonium molybdate tetrahydrate ((NH_4_)_6_Mo_7_O_24_·4H_2_O, >99.0%), copper chloride (CuCl_2_), sodium hydroxide (NaOH, >98.0%), hydrogen peroxide (H_2_O_2_, 30%), BSA, methylene blue (MB), 5,5-dimethyl-1-pyrroline *N*-oxide (DMPO), dihydroethidium (DHE), and 3,3',5,5'-tetramethylbenzidine (TMB) were purchased from Aladdin. Xanthine and xanthine oxidase, commercial colorimetric SOD activity assay kit, hydroxyl radical assay kit, 2',7'-dichlorodihydrofluorescein diacetate (DCFH-DA), lyso-tracker green (LysoTracker), rat interleukin-6 (IL-6) ELISA kit, rat tumor necrosis factor-α antibody (TNF-α) ELISA kit, rat interferon-γ (IFN-γ) ELISA kit, and calcein acetoxymethyl ester/propidium iodide (Calcein-AM/PI) were purchased from Beyotime. Antibodies including transforming growth factor-β2 antibody (TGF-β2), BCL2-associated X (Bax) antibody, CD31 antibody, and vascular endothelial growth factor antibody (VEGF) were bought from Santa Cruz (USA). Smad9 antibody, cleaved-caspase 1 antibody (C-Caspase 1), Notch3 antibody, and tumor necrosis factor-α antibody (TNF-α) were purchased from Affinity Biosciences (Jiangsu, China). α-actinin antibody and β-actin antibody were bought from Cell Signaling Technology (USA). Angiopoietin 1 polyclonal antibody (Ang1) was bought from Proteintech Group (Wuhan China). BMP8b antibody was bought from Thermo Fisher Scientific (USA). HIF-1α antibody was purchased from ZEN-BIOSCIENCE (Chengdu, China). QuickBlock blocking buffer, red blood cells, rabbit secondary antibody, mouse secondary antibody, TUNEL regent, matrigel, hematoxylin & eosin (H&E), masson reagent, Dulbecco's modified eagle medium (DMEM), fetal bovine serum (FBS), and penicillin-streptomycin mixture were purchased from Solarbio. H9C2 cells and mouse cardiac microvascular endothelial cells (MCMECs) were purchased from Procell Life Science & Technology Co., Ltd.

### Characterizations

Transmission electron microscopy (TEM) images were obtained by a HT7700-MS2 transmission electron microscope (HITACHI, Japan) operated at an accelerating voltage of 100 kV. Powder X-ray diffraction (XRD) patterns were characterized by a Shimadzu XRD-6000 diffractometer (SHIMADZU, Japan) with Cu Kα radiation. Atomic force microscopy (AFM) measurements were performed in tapping mode using a MultiMode 8 system (Bruker, USA) to determine the sample thickness. Electron spin resonance (ESR) spectra were acquired with an EMX1598 spectrometer (Bruker, USA). X-ray photoelectron spectroscopy (XPS) analysis was conducted by an Escalab 250Xi spectrometer equipped with an Al Kα X-ray source (Thermo Scientific, USA). The sizes and zeta potentials were determined by dynamic light scattering (DLS) on a Zetasizer instrument (Malvern, UK). The concentration of Co was quantified by inductively coupled plasma-atomic emission spectrometry (ICP-AES, SHIMADZU, Japan). For biological evaluations, an OGD/R model was established using a hypoxia incubator chamber (Billups-Rothenberg, USA). Fluorescence images were captured using a Nikon ECLIPSE Ti microscope (Nikon, Japan). Protein expression levels were analyzed by western blotting, and the chemiluminescent signals were detected with a ChemiDic^TM^ XRS+ Imaging System (Bio-Rad, USA).

### Preparation of ZnSrMo-LDH nanosheets

ZnSrMo-LDH nanosheets were prepared using a hydrothermal method. Briefly, solution A was prepared by dissolving Zn(NO_3_)_2_·6H_2_O (3.2 mmol), Sr(NO_3_)_2_ (0.8 mmol), and (NH_4_)_6_Mo_7_O_24_·4H_2_O (0.714 mmol) in 70 mL of deionized water under continuous stirring. Subsequently, a NaOH solution (40 mg·mL^-1^) was added dropwise to solution A under a nitrogen (N_2_) atmosphere until the pH reached 9.8. The mixture was then vigorously stirred for 30 min at room temperature to ensure homogeneity. The prepared suspension was transferred into a Teflon-lined stainless-steel autoclave and exposed to hydrothermal crystallization at 80 °C for 24 h. The precipitate was harvested after cooling to room temperature, washed thoroughly with deionized water and ethanol, and then dried for additional analysis.

### Preparation of ZnSrMo-LDH/Cu nanosheets

Briefly, ZnSrMo-LDH nanosheets (10 mg) were mixed into 10 mL of solution that included 10 mg of CuCl_2_ and 200 mg of polyvinylpyrrolidone (PVP) under stirring. The mixture was then subjected to vigorous agitation in an oil bath at 80 °C for 10 min. The resulting ZnSrMo-LDH/Cu was collected by centrifugation and washed thoroughly three times with deionized water to remove residual reactants. The final product was dried under vacuum for further use.

### Preparation of ZnSrMo-LDH/Cu-BSA nanosheets

BSA (200 mg) was added to the ZnSrMo-LDH/Cu dispersion (10 mg·mL^-1^, 10 mL) under stirring. After stirring the mixture at room temperature for 12 h, the final product was obtained through centrifugation, washed three times with deionized water, and vacuum-dried for subsequent characterization and application.

### Evaluation of ·O_2_^-^ scavenging by ZnSrMo-LDH/Cu

The ·O_2_**^-^** scavenging capacity of ZnSrMo-LDH/Cu was evaluated using a DHE fluorescent assay. Briefly, 1 mM phosphate-buffered saline (PBS) solution (pH 7.0) with 0.5 mM xanthine, 20 mU·mL^-1^ xanthine oxidase, 0.1 mM diethylenetriamine penta-acetic acid (DTPA), and ZnSrMo-LDH/Cu (100 μg·mL^-1^) or Fe_3_O_4_ (100 μg·mL^-1^) were mixed with 20 μM DHE. The fluorescent intensity was measured using a fluorophotometer with excitation/emission wavelengths of 510/580 nm. ESR spectroscopy with DMPO as a probe was further utilized to confirm the ·O_2_**^-^** scavenging capacity of ZnSrMo-LDH/Cu through similar steps.

### Evaluation of enzyme-like activity

The SOD-like activity of ZnSrMo-LDH/Cu was quantitatively assessed using a commercial SOD activity assay kit (S311-10, Dojindo Molecular Technologies, USA). The samples (ZnSrMo-LDH/Cu, ZnSrMo-LDH/Cu-BSA or Fe_3_O_4_, 100 μg·mL^-1^) were incubated with the reaction mixture containing water-soluble tetrazolium salt (WST-1), which competitively reacts with the ·O_2_^-^ generated by the xanthine oxidase system. The enzyme activity was assessed by reading the absorbance at 450 nm with a microplate reader. The SOD-like activity of ZnSrMo-LDH/Cu was further determined by monitoring the inhibition of nitrotetrazolium blue chloride (NBT) reduction. In a typical assay, the solution containing NBT (0.2 mM), xanthine (0.3 mM), and xanthine oxidase (0.1 U/mL) was mixed with different concentrations of ZnSrMo-LDH/Cu (5-200 μg·mL^-1^) in Tris-HCl buffer (0.1 M, pH 7.4). The absorbance at 560 nm was continuously monitored within 10 min.

### Evaluation of H_2_O_2_ consumption capacity of ZnSrMo-LDH/Cu

The H_2_O_2_-scavenging capacity of ZnSrMo-LDH/Cu was assessed using a titanium sulfate colorimetric method. Briefly, ZnSrMo-LDH/Cu (5-200 μg·mL^-1^) was incubated with H_2_O_2_ (1 mM) in a pH=5.4 buffer solution for 30 min. Subsequently, titanium sulfate (TiSO_4_) solution was added to form a yellow [Ti(O_2_)]^2+^ complex. After centrifugation, the supernatant was collected, and its absorbance was measured at 405 nm. The H_2_O_2_ scavenging efficiency was calculated by comparing the absorbance between the experimental and control groups.

### *In vitro* free radical detection

The ·OH generation capacity of ZnSrMo-LDH/Cu was evaluated using TMB assay. Typically, TMB (200 μL, 8 mM) and H_2_O_2_ (20 μL, 100 mM) were mixed with ZnSrMo-LDH or ZnSrMo-LDH/Cu (0.2 mL, 1 mg·mL^-1^) in deionized water (1580 μL). The generated ·OH was detected by recording the absorbance of ox-TMB at 650 nm every minute.

### O_2_ generation assessment

The O_2_ generation capacity was quantitatively assessed by mixing 1.8 mL of ZnSrMo-LDH/Cu suspension (100 μg·mL^-1^), ZnSrMo-LDH/Cu-BSA (100 μg·mL^-1^) or Fe_3_O_4_ nanoparticles (100 μg·mL^-1^, control) with 200 μL of H_2_O_2_ solution (10 mM). Subsequently, the generation of O_2_ was recorded with a portable dissolved oxygen meter within 600 s.

### Sr^2+^ and Cu^2+^ release behavior

ICP-AES was employed to quantify the release profiles of Sr^2+^ and Cu^2+^ from ZnSrMo-LDH/Cu under different pH conditions. Specifically, ZnSrMo-LDH/Cu (1 mg·mL^-1^) was dispersed in 3 mL of PBS at three physiologically relevant pH values (5.4, 6.5, and 7.4). The supernatants from each pH group were collected daily by centrifugation, followed by redispersion of the ZnSrMo-LDH/Cu in 3 mL of fresh PBS at the same pH. This process was repeated until the metal ion release reached equilibrium, which occurred over a period of 28 days.

### Cell culture and treatment

H9C2 cells and MCMECs were obtained from Wuhan Pricella Biotechnology Co., Ltd. and cultured in DMEM (Gibco) supplemented with 10% FBS (Gibco) and 1% streptomycin/penicillin (Gibco) at 37 °C in a 5% CO_2_ environment.

### OGD/R model

The OGD/R model was established according to a standardized protocol. Briefly, H9C2 cells and MCMECs were cultured until reaching 80% confluence. The cells were subjected to a hypoxic environment (95% N_2_ and 5% O_2_) in glucose-free DMEM at 37 °C for 24 h to induce oxygen-glucose deprivation (OGD). For the reperfusion phase, the cells were returned to normoxic conditions (21% O_2_, 5% CO_2_) and incubated for 24 h in complete growth medium.

### *In vitro* nanozymes intervention

Following the aforementioned protocol, H9C2 cells and MCMECs were subjected to OGD treatment. Subsequently, the culture medium was replaced with 4.5 g·L^-1^ DMEM complete growth medium supplemented with ZnSrMo-LDH/Cu-BSA (20 μg·mL^-1^) or ZnSrMo-LDH-BSA (20 μg·mL^-1^), and the cells were cultured under normoxic conditions for 24 h to simulate the reperfusion.

### CCK-8 assay

H9C2 cells and MCMECs were seeded at a density of 1×10^4^ cells per well in 96-well plates and incubated for 24 h. Following either 24 h under normoxic conditions or the OGD/R procedure, the cells were washed three times with PBS. Fresh medium containing ZnSrMo-LDH/Cu-BSA at various concentrations (10-100 μg·mL^-1^) was added to each well for 24 h of incubation. Cell viability was assessed according to the CCK-8 protocol. Briefly, basal medium (100 μL) containing 10% CCK-8 reagent was added to each well and incubated at 37 °C for 2 h. The absorbance at 450 nm was measured using a microplate reader.

### Hemolysis assay

The hemocompatibility of ZnSrMo-LDH/Cu-BSA nanocomposites was evaluated using a standardized hemolysis assay. Red blood cell suspensions (10 µL) were added to 1.5 mL tubes, followed by the addition of distilled water, PBS, and ZnSrMo-LDH/Cu-BSA at concentrations of 40, 80, 120, 160, and 200 μg·mL^-1^. The mixtures were incubated at 37 °C for 3 h. The hemolysis rate was then quantified by measuring the absorbance at 540 nm using a microplate reader.

### Tube formation assay

MCMECs were seeded at 1×10^6^ cells per well in 6-well plates. After permitting full cell adhesion, the medium was removed and the cells were washed three times with PBS. The cells were subjected to OGD by exposure to a hypoxic environment in glucose-free DMEM at 37 °C for 24 h. The medium was replaced with complete growth medium containing ZnSrMo-LDH/Cu-BSA (20 μg·mL^-1^) or ZnSrMo-LDH-BSA (20 μg·mL^-1^). For reperfusion, the cells were incubated under normoxic conditions for 24 h. Subsequently, the MCMECs were trypsinized and reseeded at 1×10^5^ cells per well in 24-well plates precoated with growth factor-reduced Matrigel (300 μL per well). After 6 h of incubation at 37 °C, the cells were washed with Ca^2^⁺-free D-Hanks' balanced salt solution (HBSS). Capillary-like tube formation was observed under a microscope. The tube length and junction points were measured and averaged using ImageJ software.

### Migration assay

MCMECs were seeded at a density of 1×10^6^ cells per well in 6-well plates. The cells were subjected to OGD and scratched with a sterile pipette tip to create wounds. The medium was subsequently replaced with complete growth medium containing ZnSrMo-LDH/Cu-BSA (20 μg·mL^-1^) or ZnSrMo-LDH-BSA (20 μg·mL^-1^). The cells were allowed to recover under normoxic conditions for an additional 24 h to simulate reperfusion. Finally, wound healing was assessed under a microscope after 24 h and 48 h of incubation.

### DCFH-DA staining assay

H9C2 cells and MCMECs were seeded at 2×10^5^ cells per well in 12-well plates. The cells were subjected to OGD by exposure to a hypoxic environment in glucose-free DMEM at 37 °C for 24 h. The medium was replaced with complete growth medium containing ZnSrMo-LDH/Cu-BSA (20 μg·mL^-1^) or ZnSrMo-LDH-BSA (20 μg·mL^-1^). For reperfusion, the cells were incubated under normoxic conditions for 24 h. After reperfusion, cells were treated with DCFH-DA reagent (10 μM) in the dark and incubated in a humidified chamber at 37 °C for 20 min. Subsequently, the cells were washed three times with serum-free medium to remove excess reagent. The fluorescence signal was captured using a fluorescence microscope and analyzed via ImageJ software.

### Calcein-AM/PI staining assay

Calcein-AM/PI staining was employed to differentiate live and dead cells. H9C2 cells and MCMECs were seeded at 2×10^5^ cells per well in 12-well plates. The cells were subjected to OGD by exposure to a hypoxic environment in glucose-free DMEM at 37 °C for 24 h. The medium was replaced with complete growth medium containing ZnSrMo-LDH/Cu-BSA (20 μg·mL^-1^) or ZnSrMo-LDH-BSA (20 μg·mL^-1^). For reperfusion, the cells were incubated under normoxic conditions for 24 h. Subsequently, the cells were stained with the Calcein-AM (2 μM) and PI (5 μM) solutions in the dark at 37 °C for 20 min. Fluorescence images were captured using a fluorescence microscope and analyzed with ImageJ software.

### RNA sequencing and analysis

H9C2 cells were seeded at a density of 1×10^6^ cells per well in 6-well plates. The cells were subjected to OGD and treated during reperfusion with complete medium and ZnSrMo-LDH/Cu-BSA medium (20 μg·mL^-1^). RNA from H9C2 cells was extracted using TRIzol reagent. RNA purity and concentration were assessed with a NanoDrop 2000 spectrophotometer (Thermo Scientific, USA). The RNA libraries were prepared with the NEBNext® Multiplex RNA Library Prep Set for Illumina NovaSeq^TM^ 6000 (NEB, USA) and assessed using a Bioanalyzer 2100 (Agilent Technologies, USA). Sequencing was performed on the Illumina Novaseq^TM^ 6000 platform. Gene expression levels (FPKM) were quantified with HTSeq-count, and differential expression analysis was conducted using DESeq2. Clustering and KEGG pathway enrichment analyses were performed to identify significantly enriched pathways.

### Western blotting analysis

All cells were homogenized and lysed with BOSTER lysis buffer and protease inhibitors (Beyotime). Protein concentrations were determined using Bradford assay (Abcam). Proteins were separated by 7.5-12.5% SDS-PAGE, transferred to PVDF membranes (Bio-Rad), blocked with QuickBlock solution (Beyotime), and incubated with primary antibodies at 4 °C overnight. For H9C2 cells, primary antibodies were as follows: TGF-β2 (1:1000, sc-374658), Smad9 (1:1000, AF5114), BMP8b (1:1000, PA1-31216), Bax (1:1000, sc-7480), TNF-α (1:1000, AF7014), and β-actin (1:1000, 4967S). For MCMECs, primary antibodies were as follows: Ang1 (1:1000, 27093-1-AP), VEGF (1:1000, sc-57496), Notch3 Antibody (1:1000, AF7548), HIF-1α Antibody (1:1000, 340462), and β-actin (1:1000, 4967S). After washing three times with TBST, the membranes were incubated with goat anti-rabbit recombinant secondary antibody (1:10000, sa00001-2) or goat anti-mouse recombinant secondary antibody (1:10000, SA00001-1) at room temperature for 1 h. Signals were visualized with the ChemiDoc^TM^ XRS+ imaging system (Bio-Rad) and analyzed using ImageJ software.

### Animals and ethics statement

All animal studies were conducted in strict adherence to the National Institutes of Health (NIH) guidelines and approved by the Laboratory Animal Ethics Committee of Wenzhou Medical University. Sprague-Dawley rats (8-week-old, male) were obtained from Vital River Laboratory Animal Technology Co., Ltd (Beijing, China). The rats were housed under standard conditions with a 12/12 h light-dark cycle and given ad libitum access to water and food. The environment was maintained at 20-25 °C with 40-70% relative humidity. Euthanasia was performed using 2% isoflurane followed by cervical spine dislocation. Only male rats were used to eliminate the influence of female sex hormones.

### Establishment of MI/R model

The MI/R model was established in 8-week-old male SD rats. Anesthesia was induced with 2% isoflurane, and the rats were mechanically ventilated at 80-100 breaths per minute using a ventilator (Zhongshi Science & Technology, China). A thoracotomy was performed to expose the heart, and the left anterior descending coronary artery (LAD) was temporarily occluded with 6-0 sutures for 30 min. Reperfusion was achieved by removing the sutures. In the LDH or LDH/Cu group, 30 μL of ZnSrMo-LDH-BSA or ZnSrMo-LDH/Cu-BSA was administered via two injections (15 μL per site) into the injury border zones. The PBS group received an equivalent volume of PBS at the same sites, while no injections were given in the MI/R group. Rats in the Sham group underwent the same procedure without LAD ligation. Surgical incisions were closed with sterile 4-0 sutures. The rats were monitored for 14 days, during which their weight and mortality were recorded.

### Echocardiography

Rats were sedated at baseline, and two-dimensional echocardiographic measurements were performed using Simpson's method (IE33 digital ultrasonic scanner, Philips Medical Systems, USA) while the rats were in right lateral recumbency. Echocardiography measurements were taken before surgery (baseline) at 7 days and 14 days following MI/R. Transthoracic echocardiography assessed left ventricular (LV) dimensions, cardiac chamber size, left ventricular ejection fraction (LVEF), left ventricular fractional shortening (LVFS), left ventricular end-systolic diameter (LVIDs), and left ventricular end-diastolic diameter (LVIDd) according to the biplane modified Simpson's rule. Standard parasternal long-axis and apical chamber views were used. All measurements were conducted by a single observer who was blinded to the rats' identities, with values averaged from three cardiac cycles. Data visualization and analysis were performed using RadiAnt DICOM Viewer software.

### Inflammatory cytokine analysis

To assess the acute inflammatory response of ZnSrMo-LDH/Cu-BSA *in vivo*, 8-week-old male SD rats were injected with 30 μL of ZnSrMo-LDH/Cu-BSA (1 mg·mL^-1^) into the left ventricle. Blood samples were collected from Sham and LDH/Cu group rats after 3 days. Samples were centrifuged to isolate serum, which was subsequently analyzed using high-sensitivity ELISA kits for quantitative detection of TNF-α, IFN-γ, and IL-6 concentrations.

### Hepatic and renal function

To assess the *in vivo* biocompatibility of ZnSrMo-LDH/Cu-BSA, 8-week-old male SD rats were injected with 30 μL of ZnSrMo-LDH/Cu-BSA (1 mg·mL^-1^) into the left ventricle. Blood samples were collected after 14 days, and biochemical tests were conducted to evaluate the effects of ZnSrMo-LDH/Cu-BSA on hepatic function (ALT, AST) and renal function (UREA).

### H&E staining

To assess the long-term biocompatibility of ZnSrMo-LDH/Cu-BSA *in vivo*, 8-week-old male SD rats were injected with 30 μL of ZnSrMo-LDH/Cu-BSA (1 mg·mL^-1^) into the myocardium. After 14 days and 28 days, major organs were harvested, perfused with PBS to clear the blood, and fixed in 4% formaldehyde for paraffin embedding and sectioning. The biosafety of ZnSrMo-LDH/Cu-BSA was examined using H&E staining, with images captured using a Leica microscope.

### Complete blood count analysis

To assess the long-term biocompatibility of ZnSrMo-LDH/Cu-BSA *in vivo*, 8-week-old male SD rats received an intramyocardial injection of 30 μL of ZnSrMo-LDH/Cu-BSA (1 mg·mL^-1^). After 28 days, blood samples were collected and analyzed using an automated biochemical analyzer to assess hematological parameters.

### *In vivo* biodistribution study

Sprague-Dawley rats were administered ZnSrMo-LDH/Cu-BSA (1 mg mL^-1^, 30 µL) via intramyocardial injection, followed by euthanasia at designated time points (2, 4, 8, 16, 24, and 48 h). Major organs (heart, liver, spleen, lungs, and kidneys) were harvested and digested with concentrated nitric acid. The Zn concentration was determined by ICP-AES.

### *In vivo* pharmacokinetic study

For pharmacokinetic analysis, Sprague-Dawley rats received an intramyocardial injection of 30 μL of ZnSrMo-LDH/Cu-BSA (1 mg mL^-1^) into the left ventricular wall. Blood samples were collected at specified time points (0.5, 1, 2, 4, 6, 8, 16, 24, and 48 h), and Zn concentration in the blood was monitored by ICP-AES.

### Evans blue/TTC staining

The hearts of rats in the MI/R, PBS, and LDH/Cu groups were perfused with PBS to remove residual blood and subsequently perfused with 1% Evans blue (Solarbio). The hearts were then sectioned into 2-mm-thick slices from the apex to the base and incubated with 2% TTC (Solarbio) solution at 37 °C for 15 min in the dark. Following incubation, the slices were fixed in 4% paraformaldehyde, and images of the sections were captured using a Leica microscope. Quantitative analysis was conducted on the INF/ALV ratio of rats from the MI/R, PBS, and LDH/Cu groups.

### Histological staining

The hearts were then fixed in 4% paraformaldehyde at 4 °C for 12-24 h. Subsequently, specimens were transferred to a 30% sucrose solution (prepared with 4% paraformaldehyde) and dehydrated at 4 °C until the tissues settled. The samples were sectioned into 5-10 μm slices using a cryostat. After air-drying at room temperature for 15 min, the tissue sections were stained with Masson's trichrome staining kit (Sigma) following the manufacturer's protocol to differentiate healthy and fibrotic regions. The tissue sections were stained with DHE (5 μM) in the dark and incubated in a humidified chamber at 37 ℃ for 30 min, or stained with TUNEL according to the TdT-mediated dUTP nick-end labeling (TUNEL) kit manual (MeilunBio, China). Images were acquired using a microscope and analyzed using ImageJ software.

### Immunostaining analysis

The perfused, fixed, and cryogenically dehydrated tissue samples were cut into 5-10 μm slices. After air-drying at room temperature for 15 min, the tissue sections were stained with the indicated primary antibodies at 4 ℃ overnight. For immunohistochemistry, the sections were incubated with the following primary antibodies: C-Caspase 1 (1:200, AF4022), Ang1 (1:200, 27093-1-AP), TGF-β2 (1:200, 251481), and CD31 (1:200, sc-46694). After washing three times with PBS, the sections were incubated with goat anti-rabbit recombinant secondary antibody or goat anti-mouse recombinant secondary antibody at 37 ℃ for 1 h, and then incubated with 3,3-diaminobenzidine (DAB) and counter-stained with hematoxylin. For immunofluorescence, the sections were incubated with the following primary antibodies: TGF-β2 (1:200, 251481), Smad9 (1:200, ab262940), α-actinin (1:200, 3134S), CD31 (1:200, sc-46694). After three washes with PBS, the sections were incubated with secondary antibodies (AlexaFluor FITC, AlexaFluor TRITC, or AlexaFluor Cy5) at 37 °C for 1 h and mounted with DAPI. The slides were examined using a Nikon ECLIPSE Ti microscope (Nikon, Japan).

### Statistical analysis

Data are presented as mean ± SEM. Comparisons between two groups were performed using Student's t-test, while One-way or Two-way ANOVA followed by Tukey's multiple comparison test was used for comparisons among multiple groups. Statistical significance was defined as *p* < 0.05. Data analysis was carried out using SPSS version 19.0 and GraphPad Prism version 9.0 software.

## Results and Discussion

### Synthesis and characterization of ZnSrMo-LDH/Cu nanosheets

**Figure 2:**
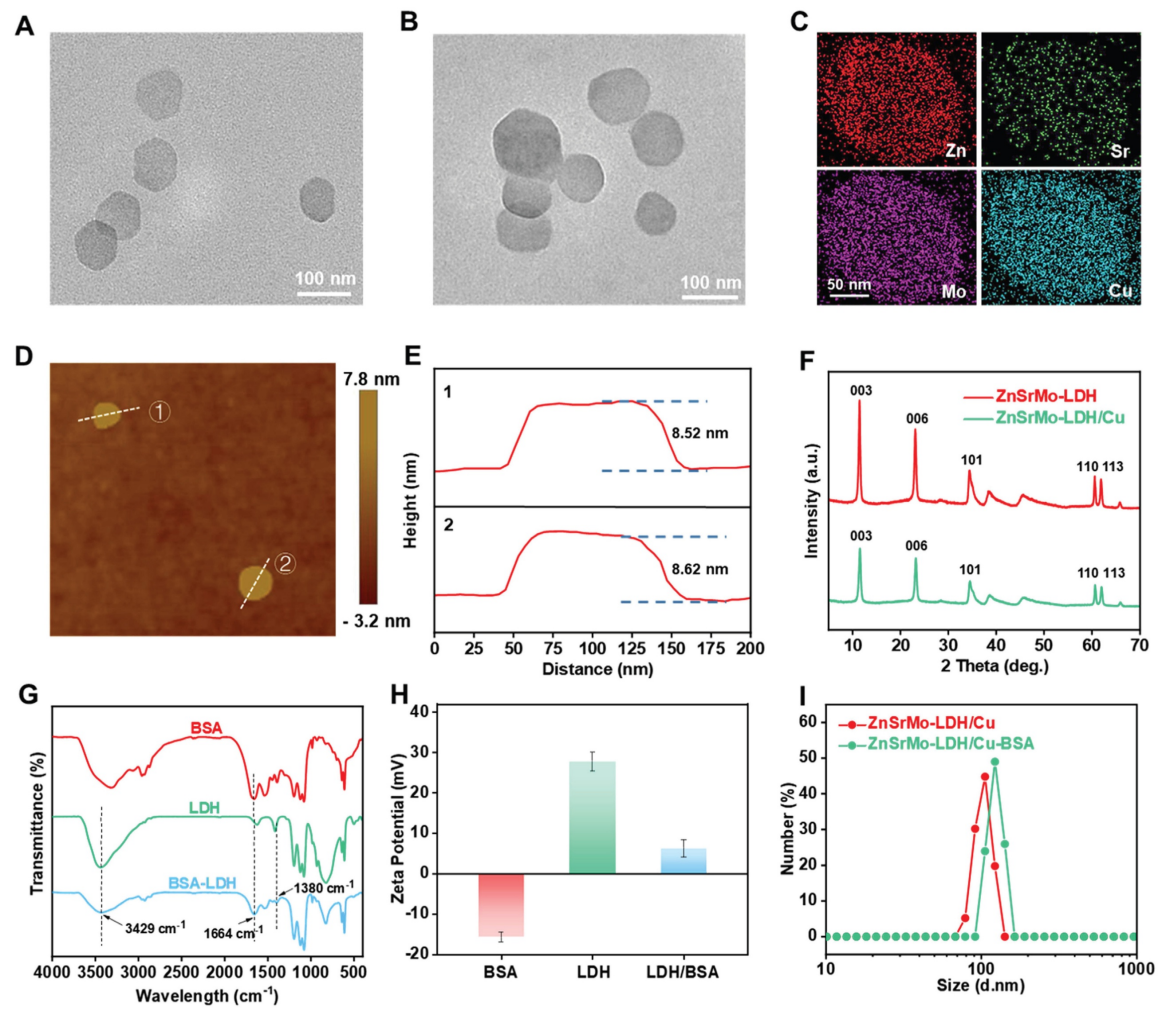
TEM images of (A) ZnSrMo-LDH and (B) ZnSrMo-LDH/Cu nanosheets. (C) EDX mapping image of ZnSrMo-LDH/Cu nanosheets. (D) AFM image and (E) corresponding height profiles of ZnSrMo-LDH nanosheets. (F) XRD patterns of ZnSrMo-LDH and ZnSrMo-LDH/Cu nanosheets. (G) FT-IR spectra of BSA, ZnSrMo-LDH/Cu, and ZnSrMo-LDH/Cu-BSA. (H) Zeta potentials of BSA, ZnSrMo-LDH, and ZnSrMo-LDH/Cu-BSA (*n* = 3). (I) The hydrodynamic sizes of ZnSrMo-LDH/Cu and ZnSrMo-LDH/Cu-BSA.

ZnSrMo-LDH nanosheets were synthesized via a low-temperature hydrothermal method, followed by the doping of Cu species through isomorphic substitution to yield ZnSrMo-LDH/Cu nanozymes. TEM analysis revealed that both ZnSrMo-LDH and ZnSrMo-LDH/Cu exhibited uniform nanosheet morphologies with similar dimensions of approximately 80-100 nm (Figure 2A and 2B). Meanwhile, the energy-dispersive X-ray (EDX) element mapping confirmed the homogeneous distribution of Zn, Sr, Mo, and Cu throughout the ZnSrMo-LDH/Cu nanosheets (Figure 2C). AFM measurements demonstrated the ultrathin 2D structure of both ZnSrMo-LDH and ZnSrMo-LDH/Cu nanosheets, with a consistent thickness of 8-9 nm (Figure 2D, 2E and S1), indicating that Cu substitution did not significantly alter the thickness of the LDH nanosheets. The XRD patterns of the ZnSrMo-LDH and ZnSrMo-LDH/Cu nanosheets displayed characteristic (003), (006), (101), (110), and (113) diffraction peaks, indicating that Cu substitutes for Zn and Sr without disrupting the crystal structure (Figure 2F). To enhance dispersion stability and biocompatibility, BSA was modified on nanosheets by electrostatic interactions. Fourier transform infrared (FT-IR) spectroscopy revealed strong absorption bands at 1664 cm^-1^ (N-H stretching vibration) of BSA, and 1380 cm^-1^ (N-O stretching) and 3429 cm^-1^ (O-H stretching) for LDH in ZnSrMo-LDH/Cu-BSA, confirming successful BSA functionalization (Figure 2G). Zeta potential measurements by DLS showed values of 27.7 ± 2.4 mV for ZnSrMo-LDH/Cu and -15.6 ± 1.2 mV for BSA. After BSA modification, the zeta potential of ZnSrMo-LDH/Cu-BSA shifted to 6.3 ± 2.1 mV (Figure 2H), which was consistent with electrostatic binding between LDH and BSA. The loading efficiency of BSA was 65.5 %, as determined by the BCA assay (Figure S2). According to DLS analysis, the hydrodynamic size of ZnSrMo-LDH/Cu-BSA had a slight change after BSA modification, with an average diameter of 122.4 ± 7.13 nm (Figure 2I). Notably, ZnSrMo-LDH/Cu-BSA maintained consistent hydrodynamic diameters within 7 days in water, PBS, and DMEM, indicating its good colloidal stability (Figure S3).

### Enzyme-mimicking antioxidant properties of ZnSrMo-LDH/Cu

**Figure 3:**
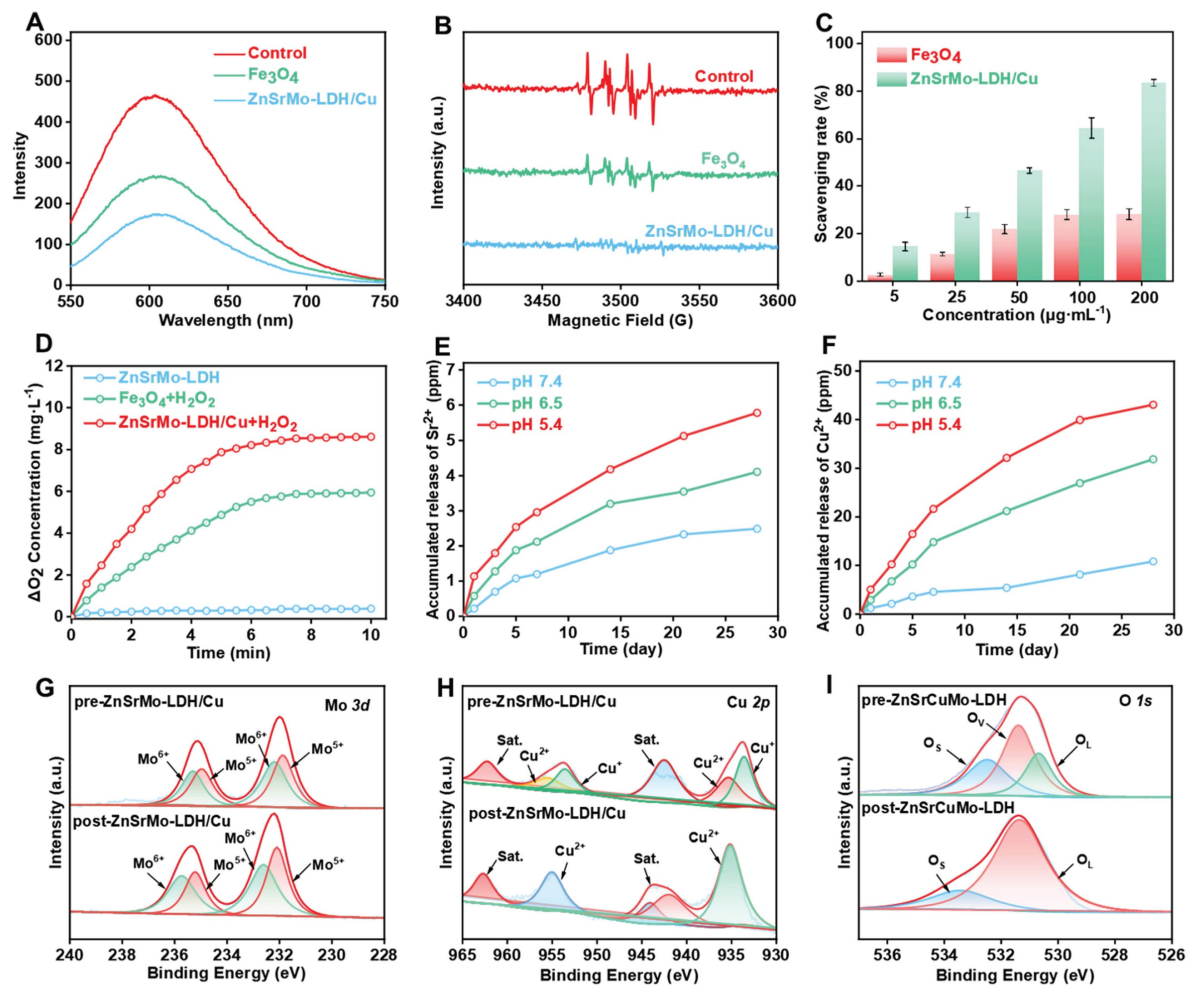
(A) The ·O_2_^-^ scavenging capacities of ZnSrMo-LDH/Cu and Fe_3_O_4_ using DHE as an indicator. (B) The ·O_2_^-^ scavenging abilities of ZnSrMo-LDH/Cu and Fe_3_O_4_ via ESR. (C) The ·O_2_^-^ scavenging capacities of ZnSrMo-LDH/Cu and Fe_3_O_4_ using SOD assay kit. (D) O_2_ generation performance of ZnSrMo-LDH/Cu and Fe_3_O_4_ in the presence of H_2_O_2_. (E) Sr^2+^ and (F) Cu^2+^ release curves of ZnSrMo-LDH/Cu under different pH buffers (5.4, 6.5 and 7.4). (G) Mo 3*d*, (H) Cu 2*p*, and (I) O 1*s* XPS spectra of pre-ZnSrMo-LDH/Cu and post-ZnSrMo-LDH/Cu.

Antioxidant nanozymes with SOD and CAT activities can effectively regulate the ROS and hypoxic microenvironments induced by sustained ischemia. Firstly, the ·O_2_^-^ scavenging ability of ZnSrMo-LDH/Cu nanozymes with different Cu contents was detected by DHE fluorescent probe to assess their SOD-mimicking activities. A progressive decrease in DHE fluorescence intensity was observed with increasing Cu content (Table S1, Figure S4), demonstrating the enhanced efficiency of Cu-mediated ·O_2_^-^ scavenging. Notably, the ZnSrMo-LDH/Cu (1:1) formulation achieved an optimized scavenging effect. Such a result was confirmed by the SOD assay, as ZnSrMo-LDH/Cu (1:1) exhibited the highest enzymatic activity (Figure S5A). Therefore, the ZnSrMo-LDH/Cu (1:1) formulation was selected for subsequent experiments. It is worth noting that ZnSrMo-LDH/Cu nanozyme significantly outperformed Fe_3_O_4_ nanozyme in ·O_2_^-^ elimination (Figure 3A), indicating its excellent antioxidant potential. Subsequently, the ·O_2_^-^ scavenging capability was further verified by ESR spectroscopy using DMPO as a spin trap. As shown in Figure 3B, the ESR signals in the ZnSrMo-LDH/Cu group almost disappeared compared with those in the Fe_3_O_4_ group, confirming the superior ability of ZnSrMo-LDH/Cu to scavenge ·O_2_^-^. Additionally, the SOD assays further revealed that ZnSrMo-LDH/Cu exhibited significantly higher SOD enzyme-like activity than Fe_3_O_4_. Specifically, at a concentration of 200 μg·mL^-1^, the SOD activity of ZnSrMo-LHD/Cu was 2.97 times that of Fe_3_O_4_ (Figure 3C). The SOD-like activity of ZnSrMo-LDH/Cu was further examined using NBT as an ·O_2_^-^ indicator. ·O_2_^-^ was generated by the xanthine/xanthine oxidase system, which reduced NBT to formazan with a strong absorbance at 560 nm. As shown in Figure S6, ZnSrMo-LDH/Cu decreased the absorbance at 560 nm in a dose-dependent manner, confirming its excellent SOD-like activity.

Given that Mo^x+^ has been shown to catalytically convert H_2_O_2_ into non-toxic O_2_ and H_2_O, we further evaluated the CAT enzyme-like activity of ZnSrMo-LDH/Cu by monitoring O_2_ production using a dissolved oxygen meter. As expected, the ZnSrMo-LDH/Cu (1:1) formulation yielded the highest O_2_ generation, demonstrating its Cu-mediated CAT-like activity (Figure S5B). As shown in Figure 3D, under the same reaction conditions, ZnSrMo-LDH/Cu exhibited stronger CAT enzyme-like activity than Fe_3_O_4_ nanozyme due to the observed higher O_2_ concentration. The H_2_O_2_-decomposition capacity of ZnSrMo-LDH/Cu was further evaluated using the titanium sulfate colorimetric assay. As depicted in Figure S7A, the absorbance at 410 nm decreased gradually with increasing ZnSrMo-LDH/Cu concentration, demonstrating dose-dependent scavenging of H_2_O_2_. This enhanced H_2_O_2_ consumption was corroborated by measurements of O_2_ generation, which rose concomitantly with higher concentrations of ZnSrMo-LDH/Cu (Figure S7B). Conclusively, ZnSrMo-LDH/Cu efficiently catalyzed the decomposition of H_2_O_2_ into O_2_, with the catalytic rate increasing proportionally with its concentration. Notably, the SOD and CAT activities of ZnSrMo-LDH/Cu-BSA are comparable to the unmodified ZnSrMo-LDH/Cu nanosheets (Figure S8), indicating that BSA modification does not significantly alter enzymatic performance. Combined with the aforementioned ·O_2_^-^ clearance ability, ZnSrMo-LDH/Cu possessed an intrinsic SOD-CAT cascade effect, which is expected to mitigate oxidative stress in cardiomyocytes and further alleviate hypoxia through cascade catalytic activity, making it a promising therapeutic agent for ischemia-related pathologies.

In addition, it is worth noting that Cu ions have been documented to display POD-like activity under acidic environments, catalyzing the generation of cytotoxic ·OH from H_2_O_2_, which is detrimental to cardiomyocyte function. In view of this, we explored the POD-like enzyme activities of ZnSrMo-LDH and ZnSrMo-LDH/Cu through TMB-based colorimetric assay. As indicated in Figure S9, compared with the ZnSrMo-LDH+H_2_O_2_ group showing negligible POD-like activity, a slight increase in TMB absorption was observed in the ZnSrMo-LDH/Cu+H_2_O_2_ group, which is attributed to the ability of introduced Cu to catalyze H_2_O_2_ into ·OH. Importantly, such a weak POD-like activity of ZnSrMo-LDH/Cu minimizes potential adverse effects on myocardial tissue. Moreover, ICP-AES measurements further demonstrated the acid neutralization ability of ZnSrMo-LDH/Cu and the responsive release performance of bioactive metal ions. In Figure 3E and 3F, Sr^2+^ and Cu^2+^ were continuously released over time under acidic conditions (pH = 5.4 and 6.5), while the amount of Sr^2+^ and Cu^2+^ detected under neutral conditions was minimal. The acid-responsive release of Sr^2+^ and Cu^2+^ is expected to enhance the targeting specificity and controllability of pro-angiogenic therapy.

To elucidate the ROS scavenging mechanism, XPS was employed to analyze valence state changes before and after ROS exposure. Zn 2*p*, Sr 3*d* and Mo 3*d* spectra of pre-ZnSrMo-LDH/Cu and post-ZnSrMo-LDH/Cu displayed similar binding energies (Figure S10). Notably, compared with pre-ZnSrMo-LDH/Cu, the Mo^5+^/Mo^6+^ ratio of post-ZnSrMo-LDH/Cu was higher after reaction with ROS (Figure 3G), demonstrating that ZnSrMo-LDH exerted CAT-like enzyme activity rather than POD-like enzyme activity. In addition, the high-resolution Cu 2*p* and O 1*s* XPS spectra of pre-ZnSrMo-LDH/Cu and post-ZnSrMo-LDH/Cu demonstrated that part of Cu^+^ ions were oxidized to Cu^2+^ ions, accompanied by the disappearance of oxygen vacancies (OVs) after ROS interaction (Figure 3H and 3I), implying that the ROS were fully adsorbed by OVs and further scavenged through Mo^5+^/Mo^6+^- and Cu^+^/Cu^2+^-mediated redox reactions.

### *In vitro* biocompatibility of ZnSrMo-LDH/Cu-BSA

The biosafety of ZnSrMo-LDH/Cu-BSA was systematically evaluated through comprehensive *in vitro* assessments. Cell counting kit-8 (CCK-8) assays were performed on H9C2 cardiomyocytes and MCMECs. The results demonstrated excellent cell viability (>90%) after 24 h of exposure to varying concentrations (10-100 μg·mL^-1^) of ZnSrMo-LDH/Cu-BSA (Figure S11A). Additionally, hemocompatibility was assessed through erythrocyte integrity testing. In Figure S11B and S11C, the hemolysis rates of red blood cells incubated with ZnSrMo-LDH/Cu-BSA (40-200 μg·mL^-1^) were always below the 5% safety threshold, meeting international standards for biomaterial compatibility. The comprehensive cytocompatibility and hemocompatibility suggest that ZnSrMo-LDH/Cu-BSA is a promising candidate for therapeutic applications.

### Cellular uptake and lysosomal escape of ZnSrMo-LDH/Cu-BSA

Efficient cellular internalization is essential for the cardioprotective efficacy of nanozymes. The uptake of FITC-labeled ZnSrMo-LDH/Cu-BSA was evaluated in both MCMECs and H9C2 cells. As shown in Figure S12A, ZnSrMo-LDH/Cu-BSA exhibited enhanced cellular uptake due to electrostatic interactions between the positively charged LDH nanostructures and the negatively charged cell membranes. Since lysosomal entrapment often limits the intracellular targeting efficiency of nanozymes, particularly for mitochondrial localization 37, we further investigated the lysosomal escape capability of ZnSrMo-LDH/Cu-BSA (Figure S12A). Confocal imaging revealed efficient lysosomal escape, which was quantitatively supported by a significantly low Pearson's colocalization coefficient (Figure S12B). These findings indicate that ZnSrMo-LDH/Cu-BSA successfully overcomes both the cellular membrane and lysosomal barriers, thereby promoting subcellular targeting and enhancing therapeutic outcomes.

### OGD/R model and optimal concentration of ZnSrMo-LDH/Cu-BSA *in vitro*

To simulate MI/R injury *in vitro*, H9C2 cells and MCMECs were exposed to OGD/R microenvironments. As shown in Figure S13, cell viability progressively decreased to 40.2% (H9C2) and 53.4% (MCMECs) after 24 h of OGD, and recovered to 64.2% (H9C2) and 65.0% (MCMECs) following 24 h of reperfusion. Consequently, we standardized the OGD/R protocol to include 24 h of OGD followed by 24 h of reperfusion for subsequent experiments, effectively mimicking the pathophysiological progression of clinical MI/R injury.

To determine the optimal concentration of ZnSrMo-LDH/Cu-BSA for protecting cells from OGD/R-induced damage, H9C2 cells and MCMECs were subjected to 24 h of OGD and then treated with varying concentrations (5-50 μg·mL^-1^) of ZnSrMo-LDH/Cu-BSA during the 24 h reperfusion period. As depicted in Figure S14, 20 μg·mL^-1^ of ZnSrMo-LDH/Cu-BSA increased the viability of H9C2 cells and MCMECs by 1.33-fold and 1.45-fold, respectively, compared to the OGD/R group. Thus, 20 μg·mL^-1^ of ZnSrMo-LDH/Cu-BSA was determined to be the optimal concentration for mitigating OGD/R injury and maintaining cellular homeostasis.

### *In vitro* antioxidant and pro-angiogenic abilities of nanosheets

**Figure 4:**
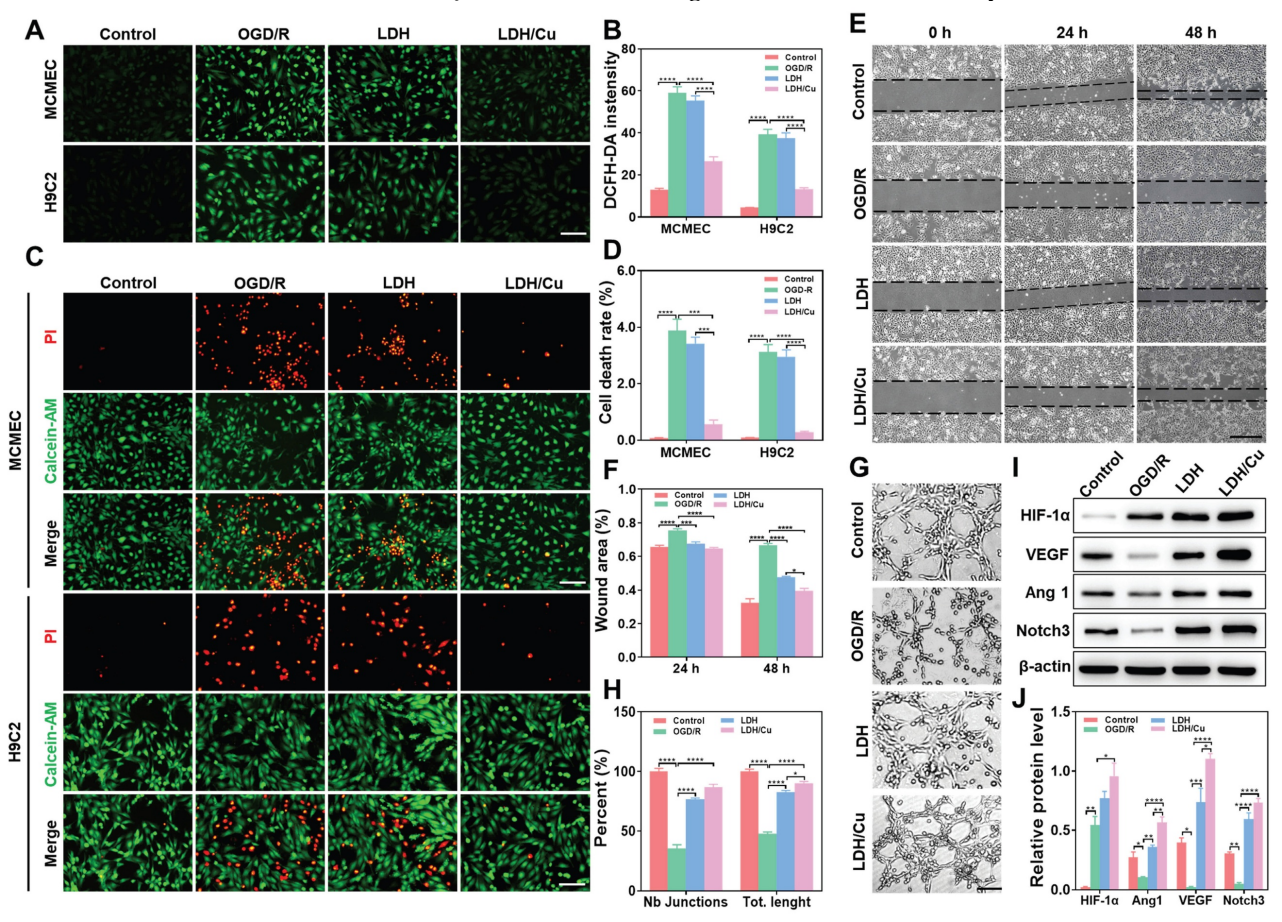
(A) DCFH-DA staining images of MCMECs and H9C2 cells after different treatments and (B) quantitative analysis of DCFH-DA fluorescence intensity, Scale bar = 125 μm (*n* = 4). (C) Calcein-AM/PI staining images of MCMECs and H9C2 cells after different treatments and (D) corresponding quantification of the cell death rates, Scale bar = 125 μm (*n* = 4). (E) Migration ability of MCMECs at 0 h, 24 h, and 48 h after different treatments and (F) corresponding quantification of wound area, Scale bar = 750 μm (*n* = 4). (G) Tube formation ability of MCMECs after different treatments and (H) corresponding quantification of tube length and junction points, Scale bar = 250 μm (*n* = 4). (I) Representative western blot bands of Ang1/VEGF/HIF-1α/Notch3/β-actin in MCMECs after different treatments and (J) corresponding quantification of relative protein levels (*n* = 3). Data are presented as mean values ± SEM, *p < 0.05, **p < 0.01, ***p < 0.001, ****p < 0.0001.

The cascade antioxidant activities of ZnSrMo-LDH-BSA and ZnSrMo-LDH/Cu-BSA were evaluated in OGD/R-injured MCMECs and H9C2 cells. Following OGD/R injury, intracellular ·O_2_^-^ and total ROS levels were markedly increased, as detected by DHE and DCFH-DA fluorescent probes. ZnSrMo-LDH-BSA treatment only marginally reduced ·O_2_^-^ and total ROS levels, whereas ZnSrMo-LDH/Cu-BSA intervention profoundly scavenged intracellular ROS (Figure 4A, 4B and S15). Moreover, apoptosis analysis via Calcein-AM/PI staining demonstrated that ZnSrMo-LDH-BSA exerted minimal effects on OGD/R-induced apoptosis in both cell types. In contrast, ZnSrMo-LDH/Cu-BSA treatment substantially reduced apoptosis in both cell types (Figure 4C and 4D). Consistent with these results, western blotting analysis further confirmed that pro-apoptotic markers (TNF-α and Bax) were significantly down-regulated after treatment with ZnSrMo-LDH/Cu-BSA compared to the OGD/R control group (Figure S16). These results collectively demonstrated that ZnSrMo-LDH/Cu-BSA exerted robust cytoprotective effects by scavenging ROS via SOD/CAT-mimetic activities and mitigating apoptosis.

Subsequently, the pro-angiogenic effects of ZnSrMo-LDH-BSA and ZnSrMo-LDH/Cu-BSA were investigated in endothelial cells. OGD/R injury significantly impaired the migratory capacity and tube formation ability of MCMECs. Interestingly, both ZnSrMo-LDH-BSA and ZnSrMo-LDH/Cu-BSA treatments effectively restored cellular motility, as evidenced by enhanced wound closure in migration assays and increased junction numbers/total length in tube formation assays (Figure 4E-H). These results suggest that the released bioactive Sr^2+^ could promote endothelial cell proliferation, migration and functionalization. ZnSrMo-LDH/Cu-BSA exhibited marginally better pro-angiogenic efficacy, likely attributable to its enhanced ROS scavenging capacity maintaining cellular viability.

To delineate the angiogenic mechanisms, we investigated alterations in the HIF-1α/VEGF/Notch signaling axis—a critical regulatory pathway for vascular formation. Western blot analysis revealed that hypoxia directly suppressed HIF-1α proteasomal degradation, causing its rapid accumulation (Figure 4I and 4J). While acute hypoxia activates angiogenic gene transcription via the HIF-VEGF-Notch pathway, sustained hypoxia disrupts downstream HIF-1α signaling, diminishing angiogenic efficiency 38,39. Persistent OGD/R impaired cellular energy metabolism, significantly reducing the expression of angiogenesis and vascular stability regulators (Ang1, VEGF, Notch3) (Figure 4I and 4J). Consistent with the migration and tube formation assays, both nanosheets treatments significantly upregulated the expression of Ang1/VEGF/Notch3. Notably, ZnSrMo-LDH/Cu-BSA demonstrated superior regulatory efficacy attributable to its exceptional ROS-scavenging capacity that promotes cellular homeostasis. These findings collectively demonstrated that ZnSrMo-LDH/Cu-BSA not only counteracted OGD/R-induced endothelial dysfunction but also actively stimulated pro-angiogenic signaling pathways. This dual action suggests substantial therapeutic potential in promoting vascular regeneration in ischemic myocardial tissue.

### *In vivo* biocompatibility of ZnSrMo-LDH/Cu-BSA

Inspired by the promising therapeutic efficacy *in vitro*, we further evaluated the therapeutic effects of ZnSrMo-LDH/Cu-BSA *in vivo*. Initially, the *in vivo* biocompatibility of ZnSrMo-LDH/Cu-BSA was investigated. A total of 30 μL of ZnSrMo-LDH/Cu-BSA solution was injected into the left ventricular myocardium. At 3 days post-administration, serum markers associated with acute inflammation were evaluated. ELISA analysis revealed no significant differences in the levels of IL-6, TNF-α, and IFN-γ compared to those in normal rats (Figure S17). At 14 and 28 days post-administration, hepatic and renal function as well as histomorphology of major organs were further assessed. Blood biochemical analysis indicated no significant differences in urea, aspartate aminotransferase (AST), or alanine aminotransferase (ALT) levels (Figure S18). H&E staining also revealed no significant morphological or microstructural alterations in major organs (heart, liver, spleen, lung, and kidney) at 14 and 28 days post-administration (Figure S19A). Analysis of the complete blood count (CBC) at 28 days post-administration confirmed that all hematological parameters remained within normal physiological ranges following the injection of ZnSrMo-LDH/Cu-BSA (Figure S19B-X). The comprehensive cytocompatibility, hemocompatibility, and systemic biocompatibility make ZnSrMo-LDH/Cu-BSA a promising candidate for therapeutic applications.

Based on the excellent biocompatibility of ZnSrMo-LDH/Cu-BSA, we further investigated its biodistribution in major organs (heart, liver, spleen, lungs, and kidneys). A total of 30 μL of ZnSrMo-LDH/Cu-BSA was administered into the left ventricular myocardium. Major organs were harvested at predetermined time points (2-48 h) and analyzed using ICP-AES. The results demonstrated that ZnSrMo-LDH/Cu-BSA exhibited prolonged accumulation in cardiac tissue, which is conducive to the repair of ischemic myocardium (Figure S20A). The blood circulation half-life of ZnSrMo-LDH/Cu-BSA was calculated to be 21.7 h through biphasic exponential fitting (Figure S20B). This prolonged circulation extends the therapeutic window, thereby enhancing the therapeutic efficacy of ZnSrMo-LDH/Cu-BSA.

### *In vivo* therapeutic effects of ZnSrMo-LDH/Cu-BSA on MI/R injury

**Figure 5:**
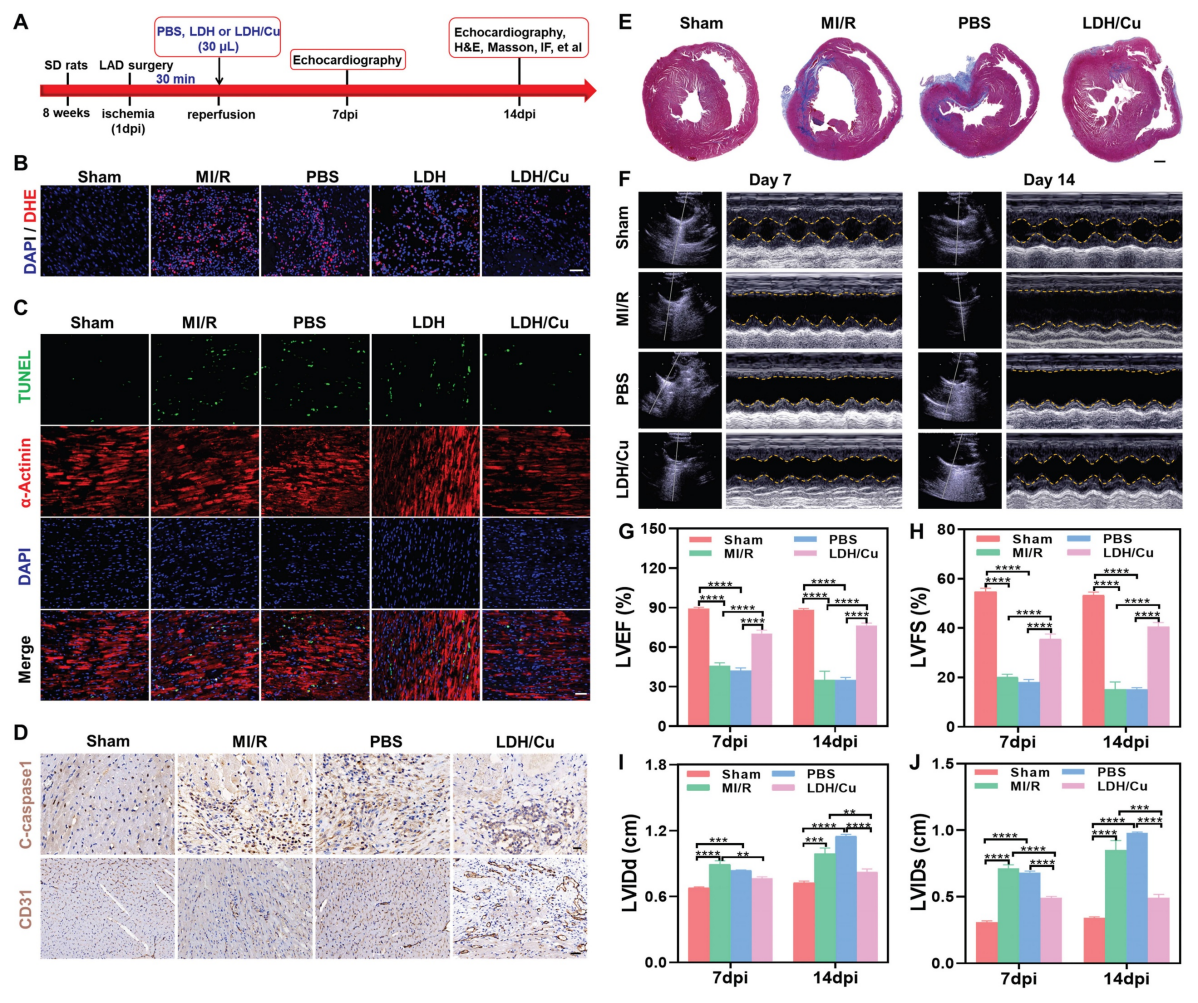
(A) Experimental timeline illustrating the treatment and analysis schedule in rats. (B) DHE staining images of myocardial tissues in SD rats after different treatments on day 14, Scale bar = 50 μm. (C) Co-immunofluorescence staining images of TUNEL (green) and α-actinin (red) of myocardial tissues after different treatments on day 14, Scale bar = 50 μm. (D) Immunostaining images of C-caspase 1/CD31 after different treatments on day 14, Scale bar (C-caspase 1) = 20 μm, Scale bar (CD31) = 50 μm. (E) Masson staining images of hearts after different treatments on day 14, Scale bar = 1000 μm. (F) Echocardiography images of cardiac function after different treatments on day 14 and quantification of ultrasonic parameters including (G) LVEF, (H) LVFS, (I) LVIDd, and (J) LVIDs (n=4). Data are presented as mean values **±** SEM, **p < 0.01, ***p < 0.001, ****p < 0.0001.

On the basis of these promising *in vitro* results, we further assessed the therapeutic effects of ZnSrMo-LDH/Cu-BSA in a rat model of MI/R injury. The experimental design followed the schedule illustrated in Figure 5A. In the LDH or LDH/Cu group, 30 μL of ZnSrMo-LDH-BSA or ZnSrMo-LDH/Cu-BSA solution was administered via two injections (15 μL per site) into the peri-infarct border zones of the infarcted myocardium immediately following reperfusion. The PBS group received equivalent PBS injections using the same delivery protocol, while the MI/R group underwent the surgical procedure without any treatment. Comprehensive histological analyses were performed at 14 days post-intervention. ROS levels in myocardial tissues were traced by the DHE assay. In Figure 5B and S21, compared with the MI/R, PBS and ZnSrMo-LDH-BSA groups, ZnSrMo-LDH/Cu-BSA significantly scavenged MI/R-induced ROS and reduced oxidative stress levels by cascading SOD/CAT-like antioxidant activities. Co-staining of α-actinin with TUNEL showed more TUNEL-labeled apoptotic cardiomyocytes in the infarcted area of the MI/R and PBS groups. The proportion of apoptotic cells was reduced in both the ZnSrMo-LDH-BSA and ZnSrMo-LDH/Cu-BSA groups (Figure 5C and S22), with more pronounced cardioprotective effects in the ZnSrMo-LDH/Cu-BSA group due to its superior ROS-scavenging capacity. Moreover, immunohistochemical staining showed lower levels of C-caspase 1 in the LDH/Cu group (Figure 5D and Figure S23A), revealing that ZnSrMo-LDH/Cu-BSA maintained myocardial redox homeostasis and inhibited ROS-induced programmed apoptosis.

According to previous reports, vascular regeneration strategies can rebuild the capillary network within the infarcted myocardium, restoring blood flow and nutrient supply. Given that ZnSrMo-LDH/Cu-BSA can respond to the acidic microenvironment to trigger the programmed release of pro-vascular Sr^2+^, we further evaluated its long-term pro-angiogenic ability *in vivo*. Immunostaining for CD31 (labeled neovascularization) was performed in the peri-infarct region on day 14 after injury. As shown in Figure 5D, S23B and S24, the ZnSrMo-LDH/Cu-BSA group conveyed more CD31 expression than the MI/R and PBS groups, demonstrating the angiogenic activity of the bioactive metal ions. Correspondingly, immunohistochemical staining showed that the expression level of Ang1 in the peri-infarct area treated with ZnSrMo-LDH/Cu-BSA was significantly increased, validating its potential to activate the pro-angiogenic signaling pathway (Figure S25). Therefore, bioactive metal ions released in response to acidity have the ability to promote vascular regeneration in the infarcted area, and newly formed capillaries can realize blood oxygen supply as early as possible.

### Cardiac function regulation by ZnSrMo-LDH/Cu-BSA

MI/R-induced oxidative stress ultimately leads to necrosis of the ischemic region and fibrotic deposition with cardiac dysfunction. Reversing the ROS microenvironment can reduce apoptosis, inhibit ventricular remodeling, and restore cardiac diastolic-systolic functions. To assess left ventricular remodeling, myocardial fibrosis in the peri-infarct region was evaluated using Masson's trichrome staining on day 14. As expected, ZnSrMo-LDH/Cu-BSA treatment significantly reduced the collagen deposition fraction and fibrotic area compared with the MI/R and PBS groups (Figure 5E and S26). To further quantify the therapeutic effect, Evans blue and 2,3,5-triphenyltetrazolium chloride (TTC) double staining were performed (Figure S27A), where blue staining indicated viable tissue, white staining marked the infarct area (INF), and TTC highlighted the area at risk (AAR). As shown in Figure S27B, the LDH/Cu group demonstrated a lower INF/area of left ventricular (ALV) ratio compared to the MI/R and PBS groups, demonstrating that ZnSrMo-LDH/Cu-BSA treatment effectively reduced cardiomyocyte apoptosis and infarction.

To thoroughly assess the effects of ZnSrMo-LDH/Cu-BSA on cardiac function and validate the clinical relevance of the aforementioned results, echocardiography was conducted on days 7 and 14 (Figure 5F). The parameters included the LVEF, LVFS, LVIDs, and LVIDd. In Figure 5G-J, the MI/R and PBS groups displayed lower LVEF and LVFS, along with higher LVIDs and LVIDd, demonstrating that severe ventricular dilatation weakened cardiac systolic function. ZnSrMo-LDH/Cu-BSA treatment could protect myocardial tissue and inhibit ventricular remodeling through antioxidant and pro-vascularization strategies, which suppressed the increase of LVIDs/LVIDd and thus improved LVEF/LVFS. Moreover, the beneficial effect of ZnSrMo-LDH/Cu-BSA on cardiac function increased with prolonged treatment. Specifically, compared to 7 days post-intervention, ZnSrMo-LDH/Cu-BSA further increased LVEF and LVFS at 14 days post-intervention (Figure 5G and 5H). These results demonstrate that ZnSrMo-LDH/Cu-BSA not only provides acute cardioprotection effects but also facilitates progressive functional recovery.

### Biological mechanisms of ZnSrMo-LDH/Cu-BSA on MI/R injury

**Figure 6:**
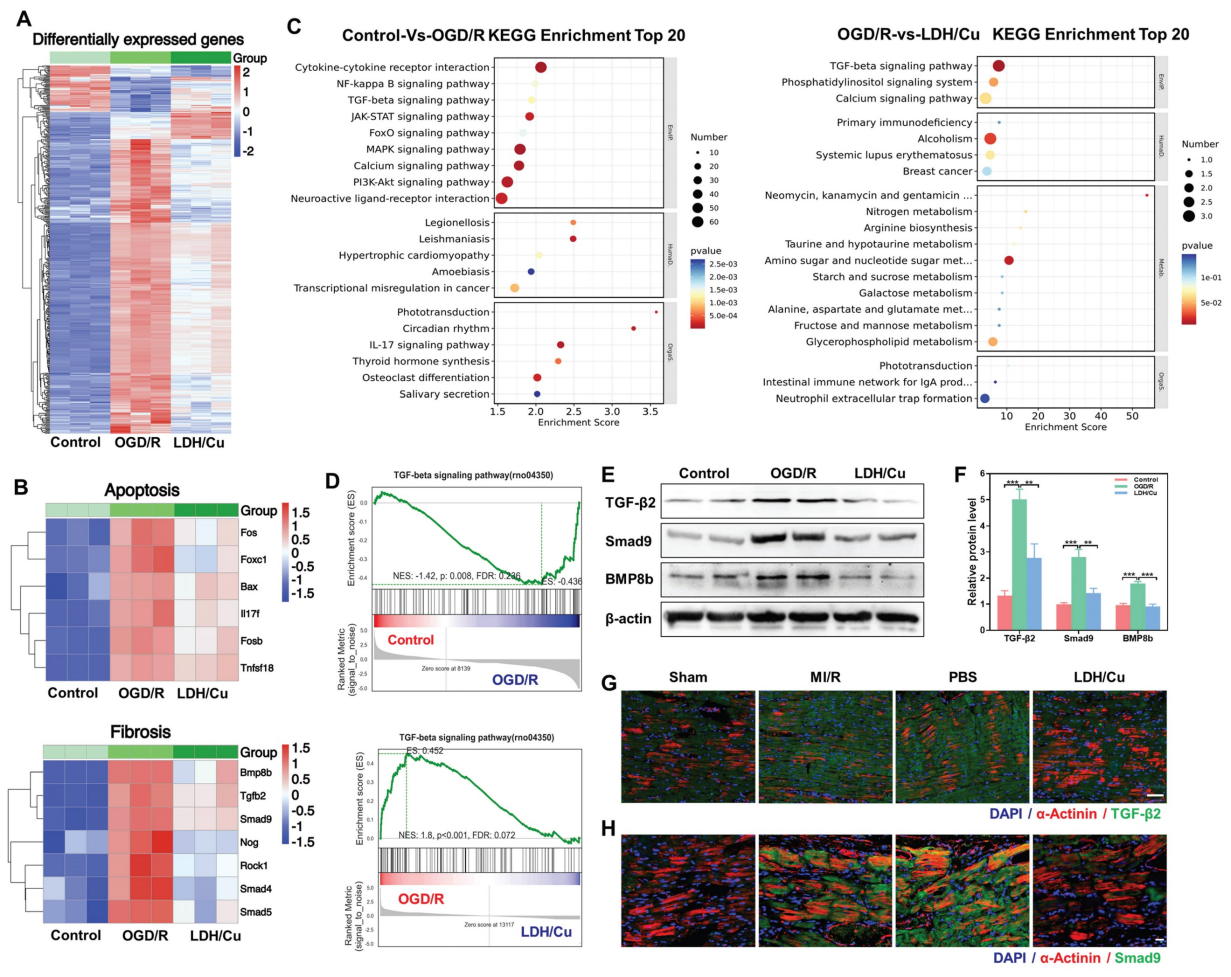
(A) Cluster analysis of total differentially expressed genes among the control, OGD/R, and LDH/Cu groups, *p* < 0.05, |Log_2_FC| >1. (B) Heatmap analysis of apoptosis-related genes and fibrosis-related genes among the control, OGD/R, and LDH/Cu groups, *p* < 0.05, |Log_2_FC| > 1. (C) KEGG enrichment analysis of total differentially expressed genes between the control and OGD/R groups, and between the OGD/R and LDH/Cu groups, *p* < 0.05, |Log_2_FC| > 1. (D) GSEA of the TGF-β signaling pathway between the control and OGD/R groups, and between the OGD/R and LDH/Cu groups, *p* < 0.05, |NES| > 1.0. (E) Representative western blot bands of TGF-β/Smad9/BMP8b/β-actin after different treatments and (F) quantification of relative protein levels (*n* = 4). (G) Co-staining images of TGF-β2 (green) and α-actinin (red) after different treatments on day 14, scale bar = 50 μm (*n* = 4). (H) Co-staining images of Smad9 (green) and α-actinin (red) of myocardial tissues after different treatments on day 14, scale bar= 20 μm (*n* = 4). Data are presented as mean values ± SEM, **p < 0.01, ***p < 0.001.

The biological mechanism of ZnSrMo-LDH/Cu-BSA for MI/R treatment was analyzed by RNA extraction and sequencing methods. Cluster analysis revealed numerous differentially expressed genes among the control, OGD/R, and LDH/Cu groups (Figure 6A). Subsequently, to assess ZnSrMo-LDH/Cu-BSA-initiated alterations in cardioprotection-related gene expression, heat map analysis was carried out, which demonstrated that ZnSrMo-LDH/Cu-BSA treatment altered gene expression profiles related to apoptosis and myocardial fibrosis, which are closely associated with myocardial infarction and ventricular remodeling (Figure 6B). To elucidate the key pathways involved, Kyoto encyclopedia of genes and genomes (KEGG) pathway analysis was performed on the differentially expressed genes. Enrichment analysis revealed that between the control and OGD/R groups, the differentially expressed genes were enriched primarily in the MAPK signaling pathway, calcium signaling pathway, PI3K/Akt signaling pathway, TGF-β signaling pathway, and cytokine-receptor interactions (Figure 6C). Following ZnSrMo-LDH/Cu-BSA intervention, the differentially expressed genes were predominantly enriched in the TGF-β signaling pathway, calcium signaling pathway, and phosphatidylinositol signaling system (Figure 6C). Additionally, gene set enrichment analysis (GSEA) indicated that the TGF-β signaling pathway, which is associated with ventricular remodeling, was significantly activated in the OGD/R group compared to the control or LDH/Cu groups (Figure 6D).

Consistent with the GSEA results, western blot analysis revealed that ZnSrMo-LDH/Cu-BSA intervention significantly attenuated the OGD/R-induced upregulation of fibrosis-related proteins (TGF-β2, Smad9 and BMP8b) in H9C2 cells (Figure 6E and 6F). Immunohistochemical staining further revealed a significant increase in TGF-β2 immunopositivity in the peri-infarct zones of the MI/R and PBS groups, which was markedly attenuated following ZnSrMo-LDH/Cu-BSA treatment (Figure S28). Furthermore, we assessed the expression levels of fibrosis-related proteins in cardiomyocytes by co-staining for α-actinin with TGF-β2 or Smad9. Compared with the LDH/Cu group, the peri-infarct areas in the MI/R and PBS groups exhibited enhanced fluorescence intensity and stronger co-localization of TGF-β2 (Figure 6G and S29) and Smad9 (Figure 6H and S30) within cardiomyocytes.

These findings demonstrate that the ROS burst during MI/R serves as a key activator of the TGF-β pathway. As a central regulator of myocardial fibrosis, TGF-β induces the transdifferentiation of fibroblasts into myofibroblasts and promotes excessive deposition of extracellular matrix (ECM), ultimately leading to cardiac structural and functional impairment 40-42. ZnSrMo-LDH/Cu-BSA nanosheets efficiently scavenge ROS through their enzymatic activity, while improving myocardial oxygen supply and nutrient delivery via pro-angiogenic effects, thereby blocking aberrant activation of the TGF-β pathway. This intervention not only mitigates myocardial injury and suppresses fibrotic progression, but also indirectly alleviates the anti-angiogenic effects of excessive fibrosis through TGF-β signaling inhibition. The tripartite synergistic mechanism of ROS scavenging, pro-angiogenesis, and TGF-β pathway suppression collectively enables enhanced myocardial repair efficacy. In summary, ZnSrMo-LDH/Cu-BSA demonstrates significant potential to enhance cardiac function and inhibit ventricular remodeling.

## Conclusion

In this work, we utilized LDHs as integrated nanocarriers to incorporate multiple bioactive metal ions into nanozymes via an isomorphic substitution strategy. ZnSrMo-LDH/Cu nanozymes possessed high ROS scavenging activity and effectively alleviated oxidative stress damage during MI/R injury through the SOD/CAT cascade. Compared with traditional nanozymes, ZnSrMo-LDH/Cu exhibited superior efficacy in scavenging ROS and converting excess ·O_2_^-^ into O_2_ and H_2_O. In addition, the hydroxide nature of LDH could neutralize tissue acidification to ensure CAT enzyme activity, accompanied by the responsive release of pro-angiogenic active ions. Both *in vitro* and *in vivo* studies unequivocally demonstrated that ZnSrMo-LDH/Cu-BSA provided excellent cardioprotective effects by modulating various biological mechanisms, including scavenging of ROS, maintenance of calcium homeostasis, inhibition of apoptotic and TGF-β signaling pathways, thereby alleviating myocardial injury caused by MI/R stimulation. Overall, this study introduces a nanoreactor with multiple antioxidant enzyme-like properties and angiogenic characteristics while minimizing the toxicity associated with traditional nanomaterials. These findings highlight the potential of ZnSrMo-LDH/Cu as a promising nanotherapeutic candidate for MI/R injury treatment.

However, the limited myocardial targeting efficiency of ZnSrMo-LDH/Cu-BSA following intravenous injection provides a compelling rationale for employing direct intramyocardial injection as the primary delivery strategy in this study. While rodent model data confirm the robust cardioprotective efficacy of ZnSrMo-LDH/Cu-BSA administered via intramyocardial injection, we acknowledge the clinical translatability challenges associated with this approach. Although intracoronary delivery demonstrates moderate improvement in cardiac drug accumulation, its clinical translation remains significantly constrained by substantial technical challenges and unacceptable procedural mortality rates. To simultaneously improve targeting efficiency and clinical applicability, future studies will focus on modifying LDH nanosheets with myocardial-targeting moieties. This modification is expected to augment the ischemia-homing capability of LDH nanosheets while maintaining the non-invasive administration advantages, thereby advancing the translational development of this nanoplatform for myocardial ischemia-reperfusion therapy.

## Supplementary Material

Supplementary figures and table.

## Abbreviations

MI/R: myocardial ischemia-reperfusion
LDH: layered double hydroxide
SOD: superoxide dismutase
CAT: catalase
POD: peroxidase
ROS: reactive oxygen species
BSA: bovine serum albumin
OGD/R: oxygen-glucose deprivation/reperfusion
NBT: nitrotetrazolium blue chloride
MB: methylene blue
DHE: dihydroethidium
Calcein-AM/PI: calcein acetoxymethyl ester/propidium iodide
TGF-β2: transforming growth factor-β2 antibody
Ang1: angiopoietin 1 polyclonal antibody
VEGF: vascular endothelial growth factor antibody
C-Caspase 1: cleaved-caspase 1 antibody
Bax: BCL2-associated X
TNF-α: tumor necrosis factor-α antibody
H&E: hematoxylin & eosin
DMEM: Dulbecco's modified eagle medium
FBS: fetal bovine serum
MCMECs: mouse cardiac microvascular endothelial cells
TEM: transmission electron microscopy
AFM: atomic force microscopy
ESR: electron spin resonance
DLS: dynamic light scattering
XO: xanthine oxidase
WST-1: water-soluble tetrazolium salt
FT-IR: Fourier transform infrared
CCK-8: Cell counting kit-8
XRD: X-ray diffraction
XPS: X-ray photoelectron spectroscopy
ICP-AES: inductively coupled plasma-atomic emission spectrometry
·OH: hydroxyl radicals
·O_2_^-^: superoxide radicals
PVP: polyvinylpyrrolidone
LV: left ventricular
LVEF: left ventricular ejection fraction
LVFS: left ventricular fractional shortening
LVIDs: left ventricular end-systolic diameter
LVIDd: left ventricular end-diastolic diameter
AST: aspartate aminotransferase
ALT: alanine aminotransferase
HGB: hemoglobin
