# Supplementary material for: Pathologically responsive ZnSrMo-LDH/Cu nanozymes with cascade antioxidant and angiogenic functions for myocardial ischemia-reperfusion treatment
Source: Theranostics. 2026 Jan 1;16(1):398–416. doi: 10.7150/thno.118420 (PMC12665128; doi:10.7150/thno.118420)
Supplement: Supplementary file 1 — Supplementary figures and table. [file thnov16p0398s1.pdf]

Supporting Information

Pathologically responsive ZnSrMo-LDH/Cu nanozymes with cascade antioxidant and angiogenic functions for myocardial ischemia-reperfusion treatment

Jian Xu[1†], Susu Zhang[1†], Yu Yang[2†], Xingwei Wei[1], Yunteng Fang[1], Zhilin Wang[1], Linwen Lan[1], Jiayi Shen[1], Enqian Liu[1], Wuming Hu[1], Tingting Hu[3,4], Chaojie Yu[3,4*], Ruizheng Liang[2,5*], and Lingchun Lyu[1*]

[1]Department of Cardiology, The Fifth Affiliated Hospital of Wenzhou Medical University, Lishui Central Hospital, Lishui, 323000, P. R. China

[2]State Key Laboratory of Chemical Resource Engineering, Beijing Advanced Innovation Center for Soft Matter Science and Engineering, Beijing University of Chemical Technology, Beijing 100029, P. R. China

[3]Department of Electrical Engineering, City University of Hong Kong, 83 Tat Chee Ave, Kowloon Tong, Hong Kong SAR 999077, P. R. China

[4]Henan Provincial People's Hospital, People's Hospital of Zhengzhou University, Zhengzhou 450003, P. R. China

[5]Quzhou Institute for Innovation in Resource Chemical Engineering, Quzhou 324000, P. R. China

[†]These authors contributed equally.

*Correspondence authors:

Chaojie Yu

E-mail: chaojie.yu@cityu.edu.hk

Ruizheng Liang

E-mail: liangrz@buct.edu.cn

Lingchun Lyu

E-mail: lvlingchun@medmail.com.cn

Table S1. Elemental contents in ZnSrMo-LDH and ZnSrMo-LDH/Cu samples.

Sample	Zn (ppm)	Sr (ppm)	Mo (ppm)	Cu (ppm)
ZnSrMo-LDH	8.66	1.65	6.39	/
ZnSrMo-LDH/Cu=1:0.5	3.64	0.71	2.86	0.36
ZnSrMo-LDH/Cu=1:1	2.48	0.54	2.33	4.9
ZnSrMo-LDH/Cu=1:2	2.16	0.51	2.15	5.05

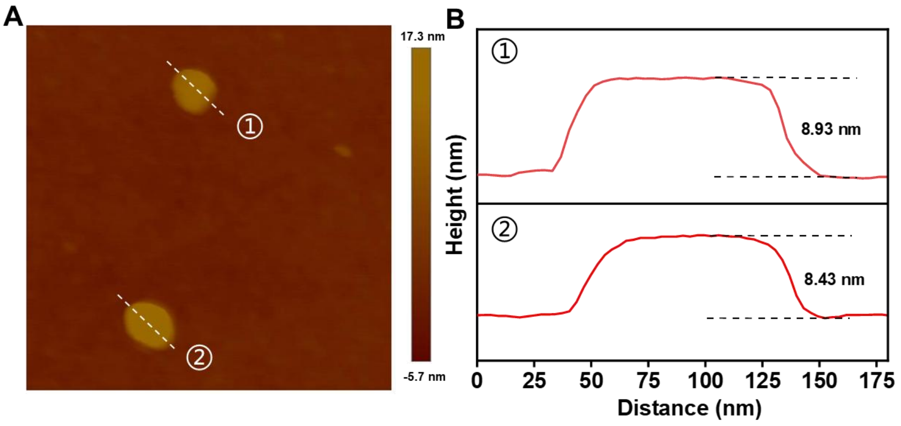

Figure S1. (A) AFM image and (B) corresponding height profiles of the ZnSrMo-LDH/Cu nanosheets.

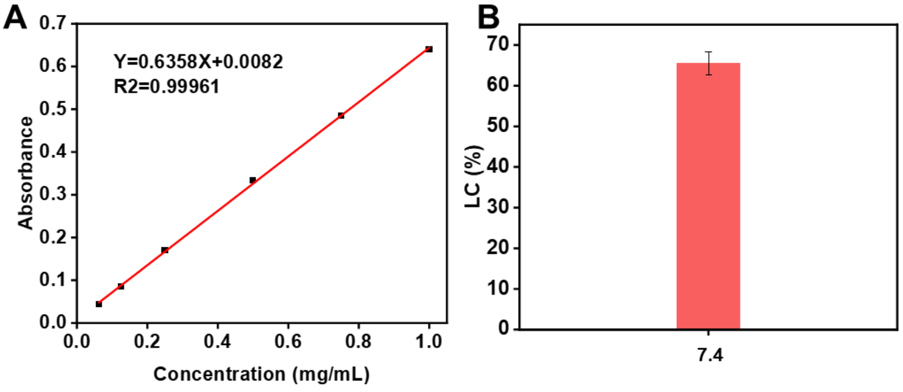

Figure S2. (A) Protein concentration standard curve. (B) BSA loading efficiency under neutral condition (pH 7.4).

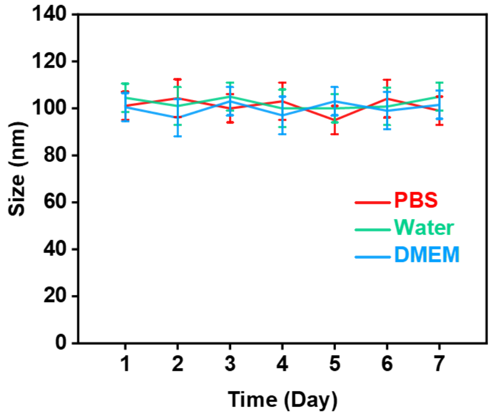

Figure S3. Stability tests of ZnSrMo-LDH/Cu-BSA in water, PBS, and DMEM. Data are presented as mean values ± S.D. (n = 3).

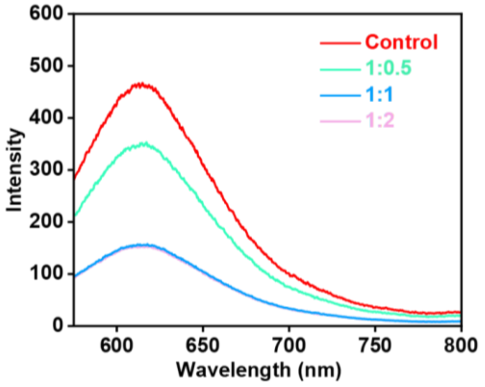

Figure S4. $\cdot O_2^-$ scavenging capacity of ZnSrMo-LDH/Cu with different Cu contents.

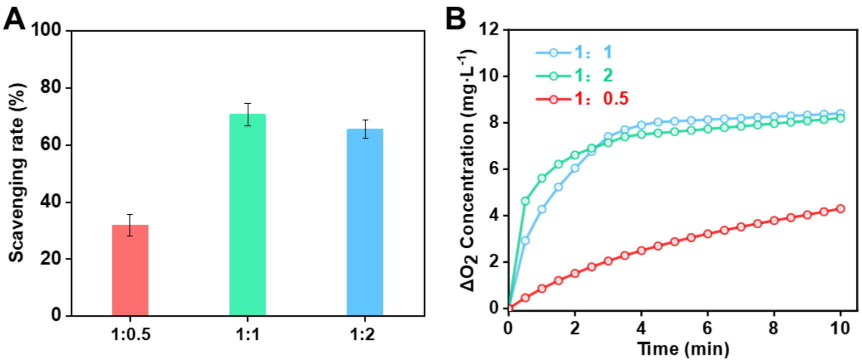

Figure S5. (A) SOD and (B) CAT enzymatic activities of ZnSrMo-LDH/Cu with different Cu contents.

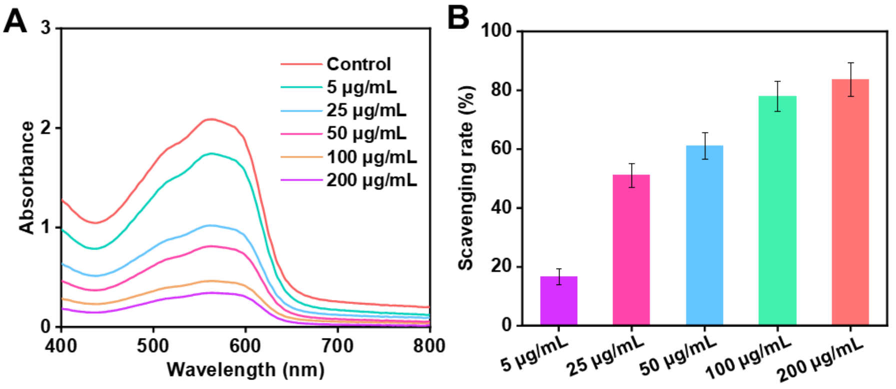

Figure S6. (A) Absorption spectra of formazan produced by the reaction with $\cdot O_2^-$ in the presence of different concentrations of ZnSrMo-LDH/Cu. (B) $\cdot O_2^-$ scavenging rates by ZnSrMo-LDH/Cu at varying concentrations.

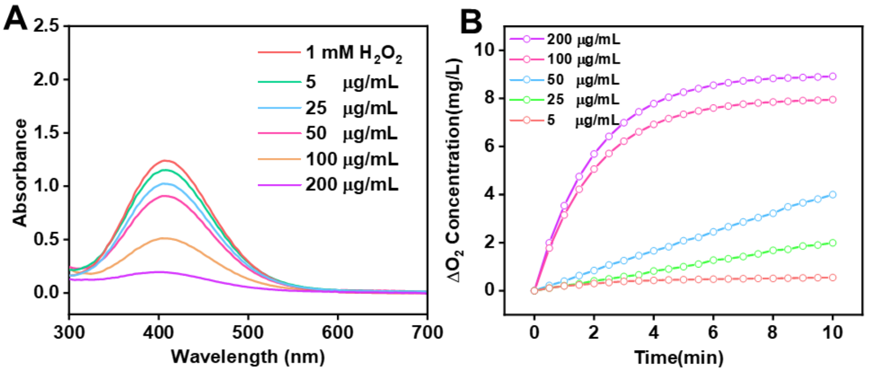

Figure S7. (A) Absorption spectra of $(TiO(H_2O_2)^{2+})$ after reaction with H_2O_2 in the presence of different concentrations of ZnSrMo-LDH/Cu. (B) O_2 generation catalyzed by ZnSrMo-LDH/Cu via decomposition of H_2O_2.

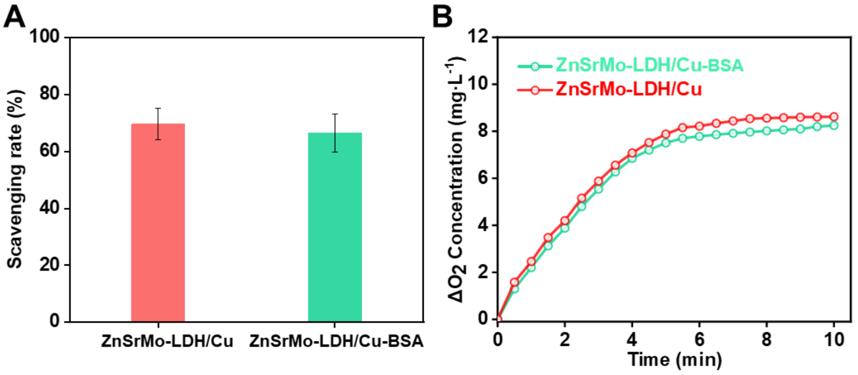

Figure S8. (A) SOD and (B) CAT enzymatic activities of ZnSrMo-LDH/Cu and ZnSrMo-LDH/Cu-BSA nanosheets.

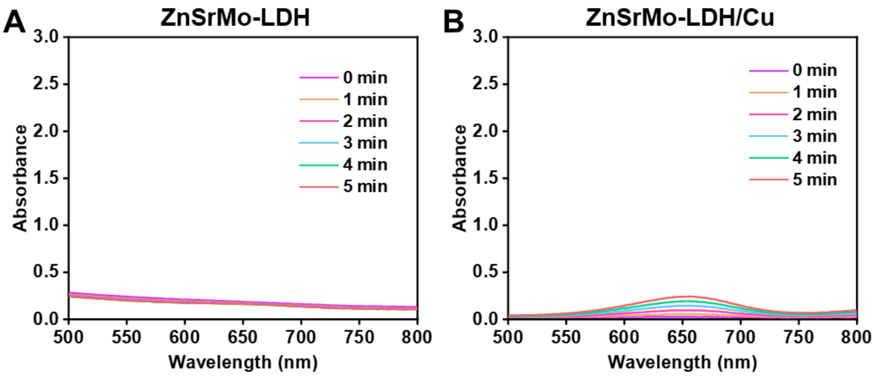

Figure S9. UV-vis absorption spectra of ox-TMB in the presence of (A) ZnSrMo-LDH+H_2O_2 and (B) ZnSrMo-LDH/Cu+H_2O_2, respectively.

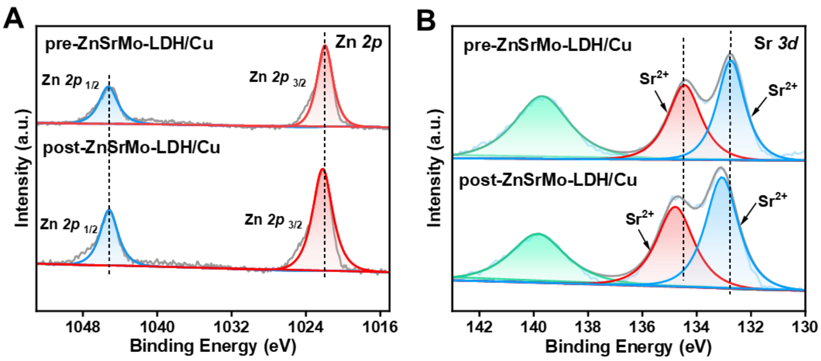

Figure S10. XPS Zn 2*p* (A) and Sr 3*d* (B) spectra of pre-ZnSrMo-LDH/Cu and post-ZnSrMo-LDH/Cu.

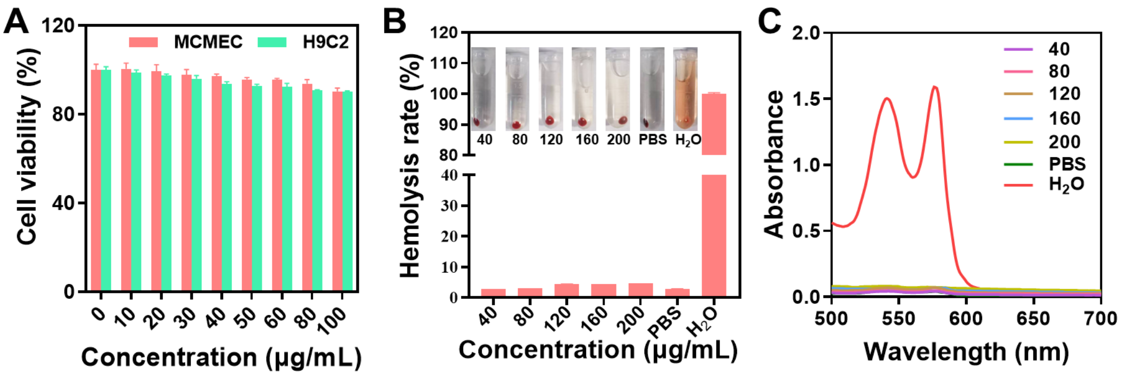

Figure S11. (A) Cell viability of MCMECs and H9C2 cells cultured with ZnSrMoLDH/Cu-BSA at different concentrations (n = 4). (B) Hemolysis rates of red blood cells after incubation with different concentrations of ZnSrMoLDH/Cu-BSA (n = 3). Inset: representative photographs. (C) Absorbance of red blood cell supernatant treated with H_2O, PBS and ZnSrMoLDH/Cu-BSA at different concentrations (n = 3). Data are presented as mean values ± SEM.

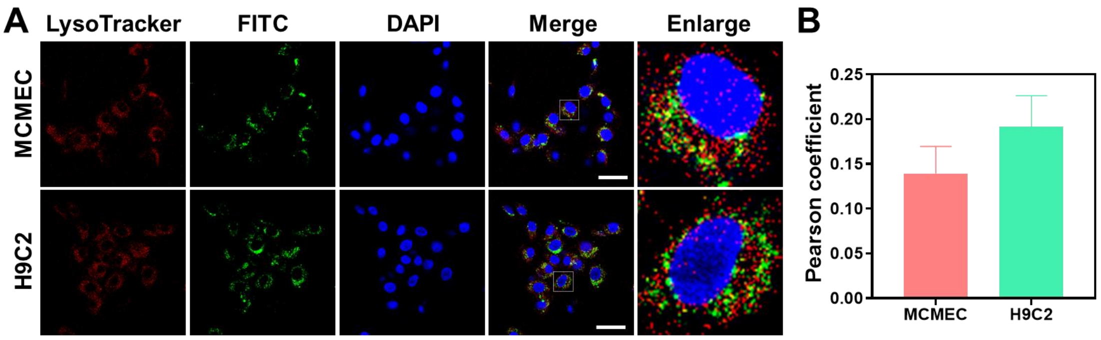

Figure S12. (A) Colocalization of FITC-labeled ZnSrMoLDH/Cu-BSA with lysosomes and (B) quantification analysis of the Pearson's colocalization coefficient, Scale bar = 40 μm (n = 3). Data are presented as mean values ± SEM.

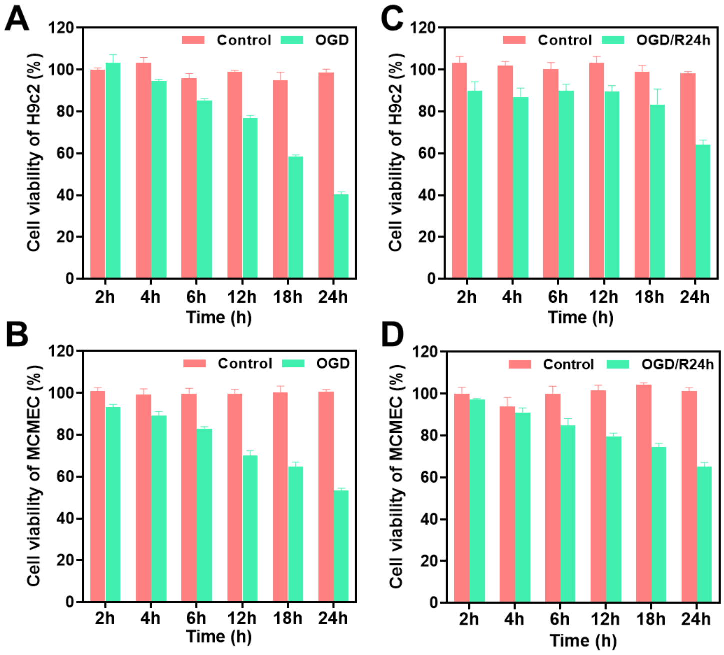

Figure S13. (A, B) Cell viability of H9C2 cells and MCMECs after 2~24 h of exposure to OGD. (C, D) Cell viability of H9C2 cells and MCMECs after 2~24 h of exposure to OGD/R. Data are presented as mean values ± SEM (n = 6). Data are presented as mean values ± SEM.

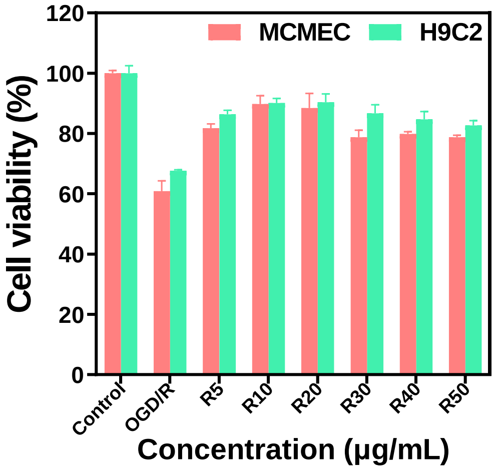

Figure S14. Cell viability of H9C2 cells and MCMECs after different treatments (n = 4). Data are presented as mean values ± SEM.

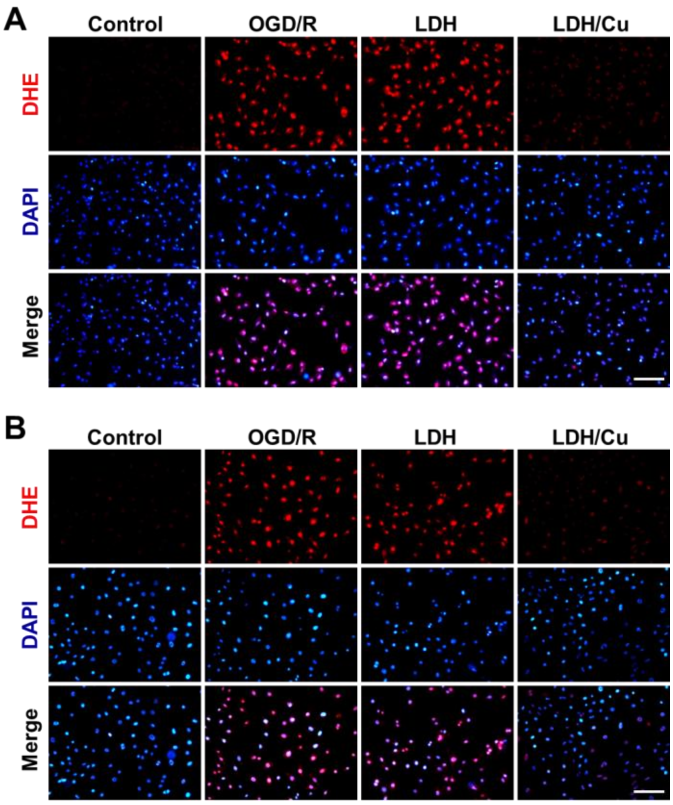

Figure S15. (A) DHE staining images of H9C2 cells and (B) MCMCEs after different treatments, Scale bar = 125 μm (n = 4).

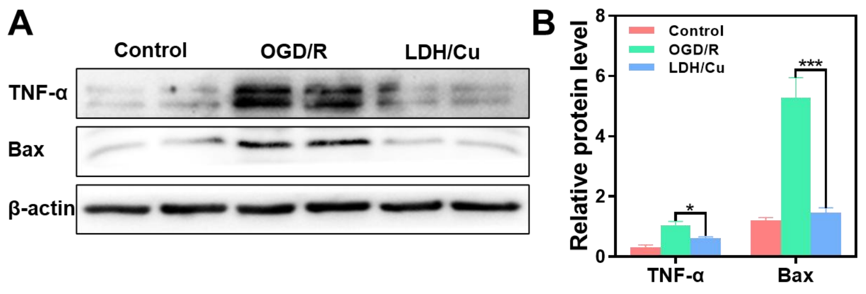

Figure S16. (A) Representative western blot bands of TNF-α/Bax/β-actin after different treatments and (B) quantification of relative protein levels ($n = 4$). Data are presented as mean values ± SEM, *$p < 0.05$, *** $p < 0.001$.

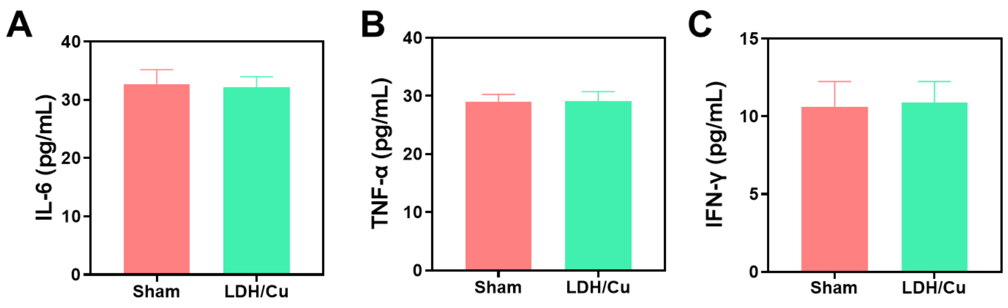

Figure S17. Inflammatory cytokine levels of SD rats detected after injection of ZnSrMoLDH/Cu-BSA on day 3 ($n = 5$). Data are presented as mean values ± SEM.

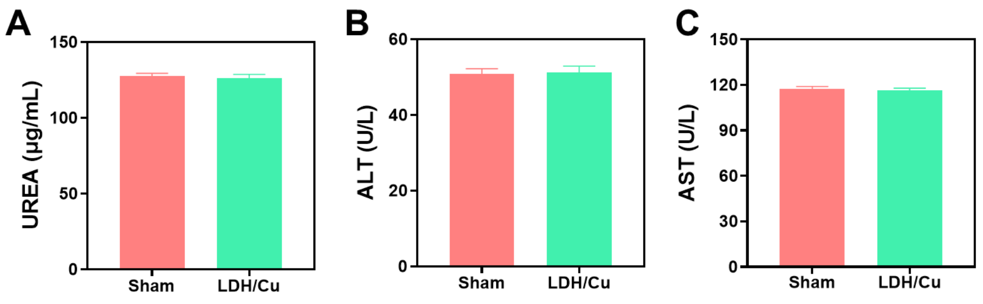

Figure S18. Liver and kidney function indexes after SD rats injection with ZnSrMoLDH/Cu-BSA on day 14 ($n = 3$). Data are presented as mean values ± SEM.

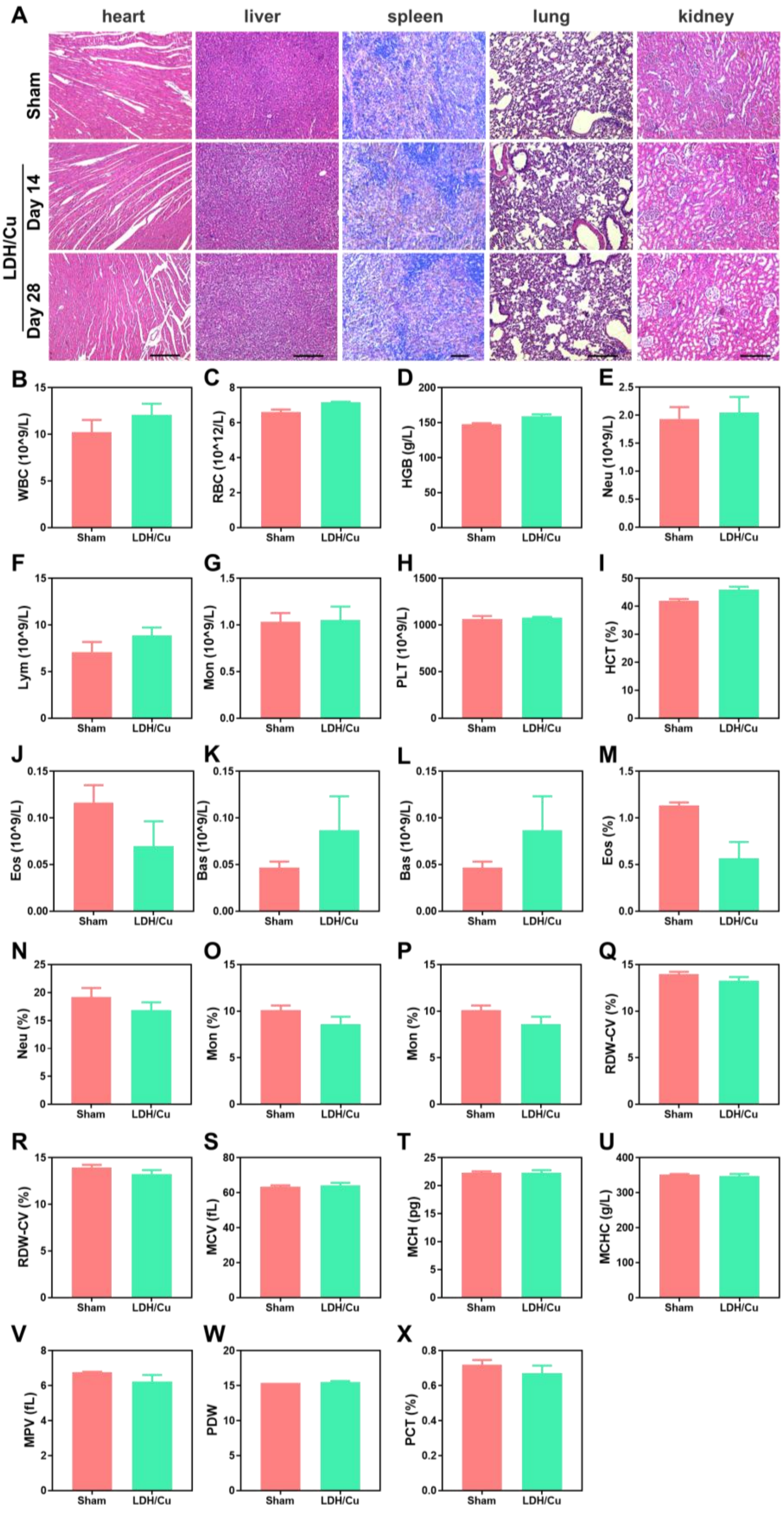

Figure S19. (A) H&E staining images of the heart, liver, spleen, lung, and kidney after injection of ZnSrMoLDH/Cu-BSA on day 14 and day 28, Scale bar (heart, liver, lung, kidney) = 100 μm, scale bar (spleen) = 75 μm (n = 5). (B-X) Complete Blood Count (CBC) of SD rats detected on day 28 after injection of ZnSrMoLDH/Cu-BSA (n = 3). Data are presented as mean values ± SEM.

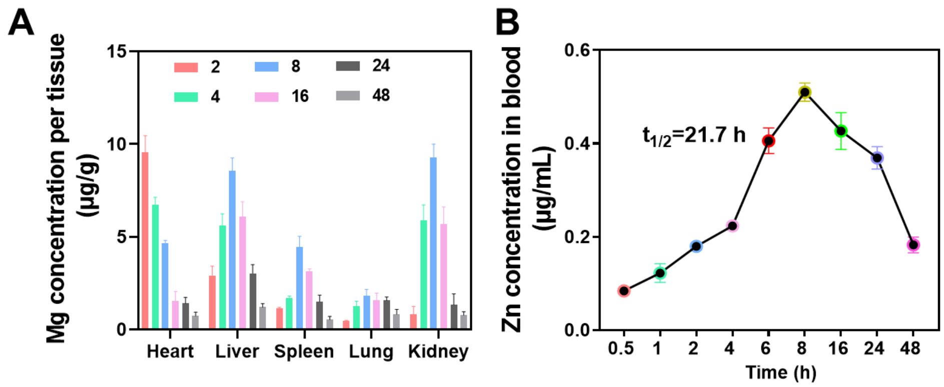

Figure S20. (A) Biodistribution and (B) pharmacokinetic profile of ZnSrMoLDH/Cu-BSA in rats by monitoring Zn concentration at various time points post intramyocardial injection (n = 3). Data are presented as mean values ± SEM.

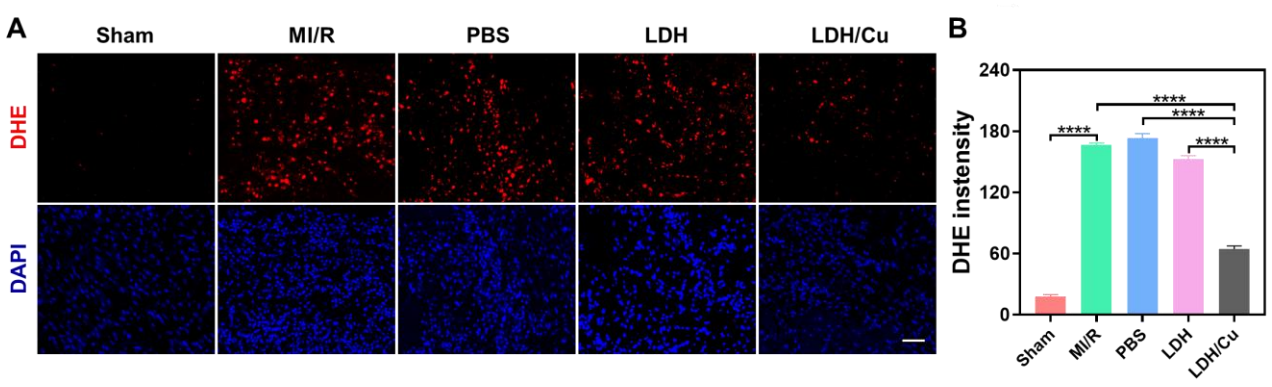

Figure S21. (A) Single-channel images from Figure 5B and (B) quantification of fluorescence intensity, scale bar = 50 μm (n = 6). Data are presented as mean values ± SEM, ****p < 0.0001.

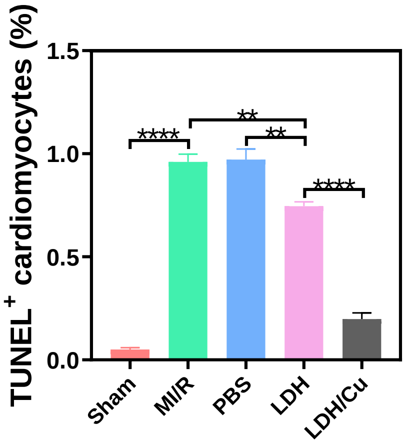

Figure S22. Quantification of apoptosis by TUNEL-positive expression from Figure 5C. Data are presented as mean values ± SEM (n = 6), **p < 0.01, ****p < 0.0001.

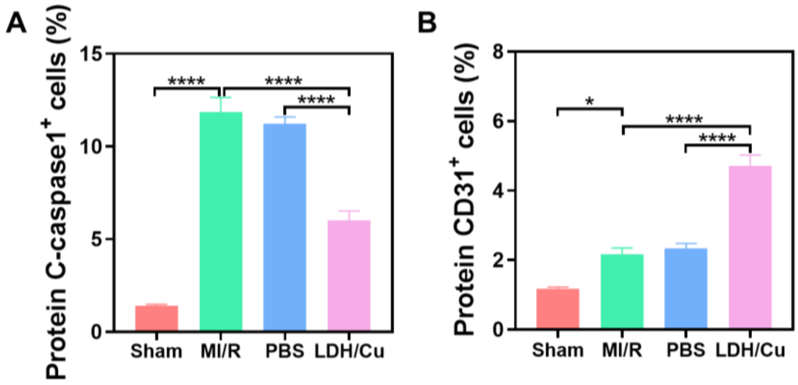

Figure S23. Quantitative analysis of the relative expression of (A) C-caspase1 and (B) CD31 from Figure 5D (n = 6). Data are presented as mean values ± SEM, *$p < 0.05$, ****$p < 0.0001$.

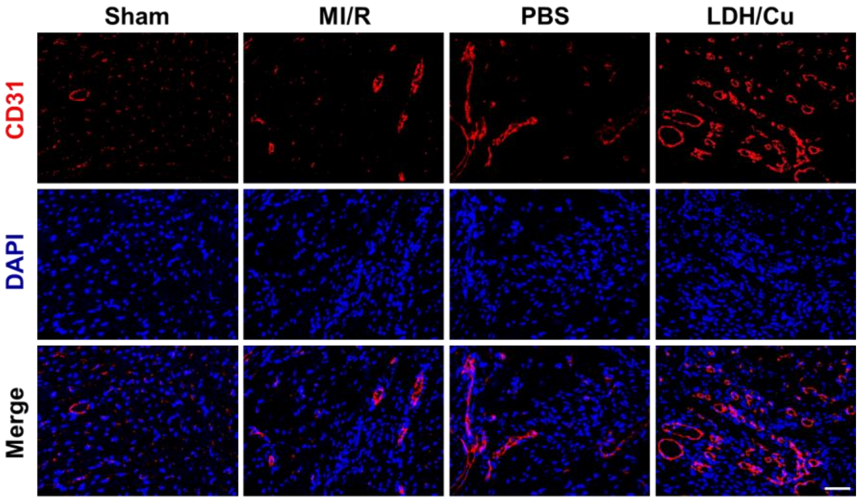

Figure S24. Immunofluorescence images of CD31 after different treatments on day 14, scale bar = 50 μm (n = 5).

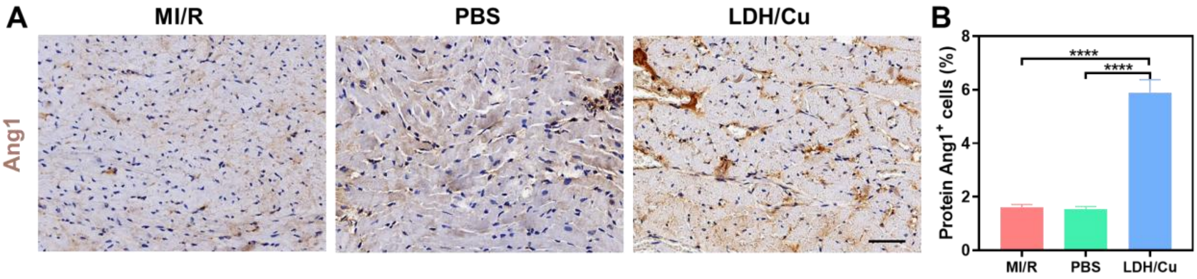

Figure S25. (A) Immunohistochemical staining images of Ang1 after different treatments on day 14 and (B) relative protein expression of Ang1, scale bar = 50 μm (n = 6). Data are presented as mean values ± SEM, ****$p < 0.0001$.

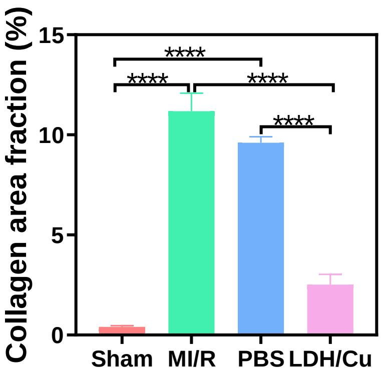

Figure S26. Collagen area fraction after different treatments on day 14 from Figure 5E ($n = 3$). Data are presented as mean values ± SEM, ****$p < 0.0001$.

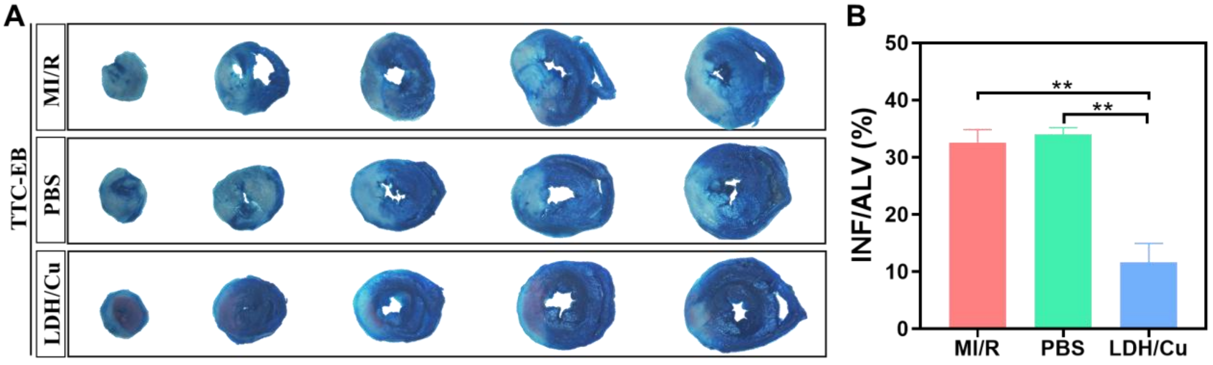

Figure S27. (A) TTC-Evans blue staining images of myocardial tissue slices (thickness~2 mm) from apex (left) to base (right) and (B) quantitative analysis of INF/ALV ratio ($n = 3$). Data are presented as mean values ± SEM, ** $p < 0.01$.

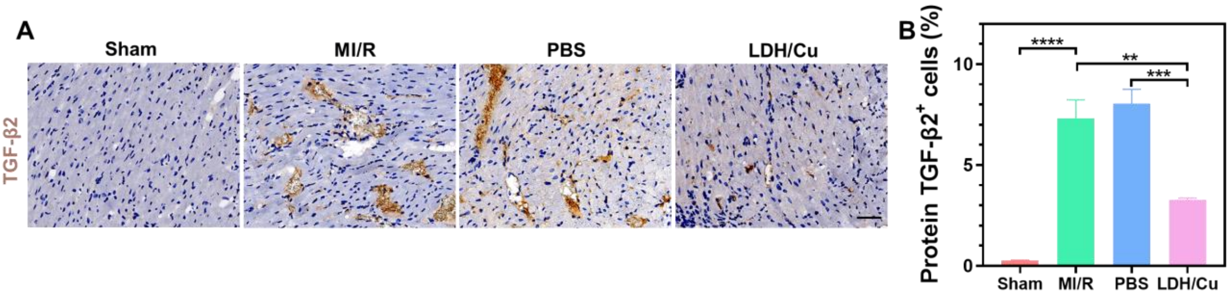

Figure S28. (A) Immunostaining images of TGF-β2 after different treatments on day 14 and (B) quantification of protein levels, Scale bar = 50 μm ($n = 4$). Data are presented as mean values ± SEM, **$p < 0.01$, ***$p < 0.001$, ****$p < 0.0001$.

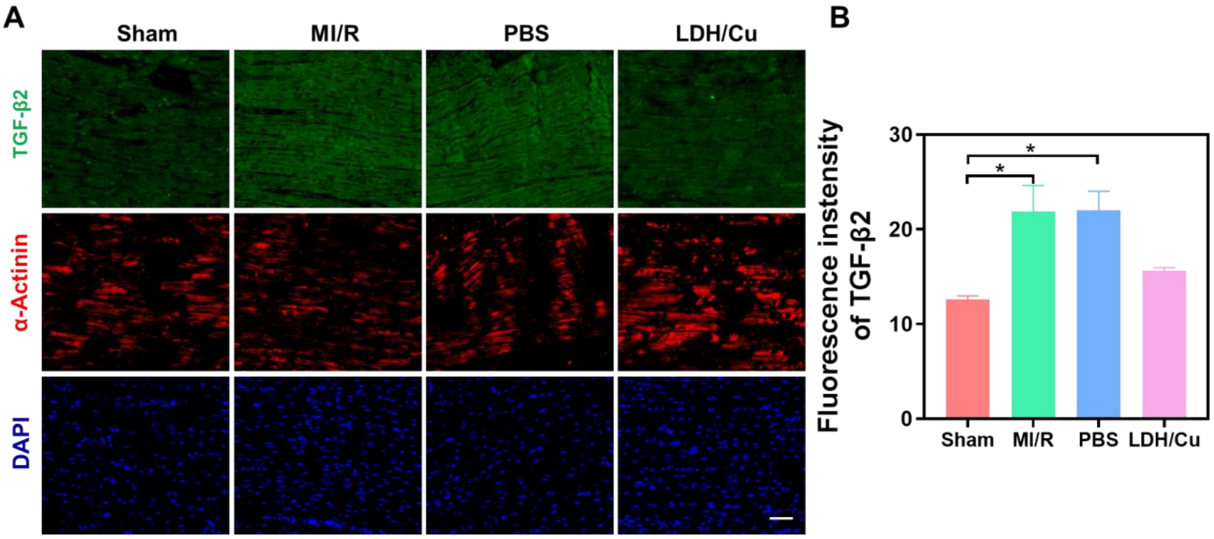

Figure S29. (A) Single-channel images of TGF-β2 (green) and α-actinin (red) after different treatments on day 14 from Figure 6G and (B) quantification analysis of the fluorescence intensity, Scale bar = 50 μm (n = 4). Data are presented as mean values ± SEM, *$p < 0.05$.

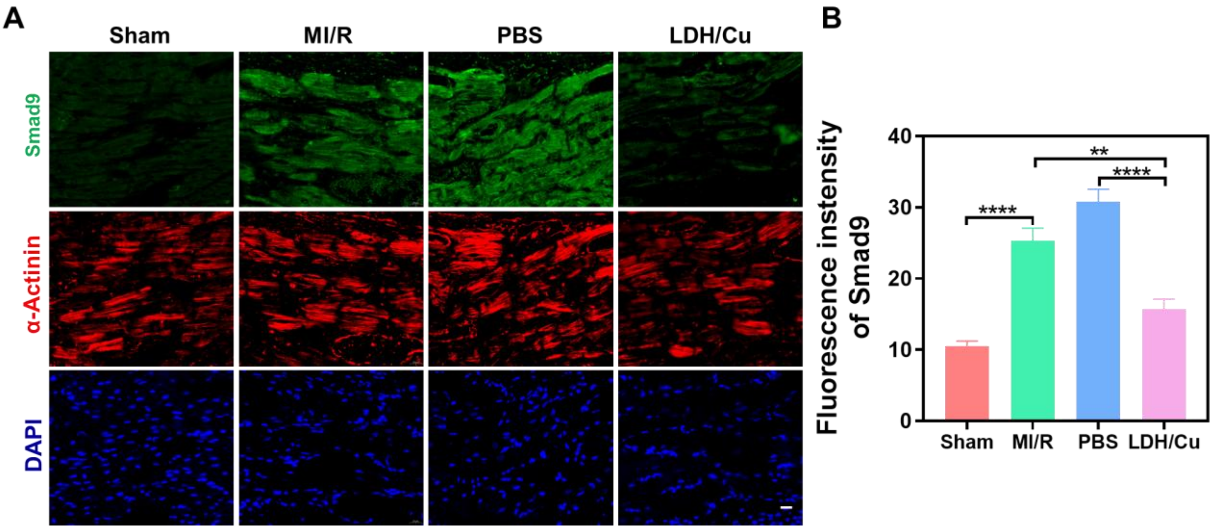

Figure S30. (A) Single-channel images of Smad9 (green) and α-actinin (red) after different treatments on day 14 from Figure 6H and (B) quantification analysis of the fluorescence intensity, Scale bar = 20 μm (n = 4). Data are presented as mean values ± SEM, **$p < 0.01$, ****$p < 0.0001$.
